# Geschichte der deutschsprachigen HNO-Zeitschriften

**DOI:** 10.1007/s00106-021-01035-y

**Published:** 2021-04-07

**Authors:** Norbert Stasche, Michael Bärmann

**Affiliations:** 1 Medizinische Fakultät Mannheim, Universität Heidelberg, Theodor-Kutzer-Ufer 1–3, 68167 Mannheim, Deutschland; 2 HNO-Klinik, Westpfalz-Klinikum GmbH, Hellmut-Hartert-Str. 1, 67655 Kaiserslautern, Deutschland

**Keywords:** Medizinjournalismus, Zeitschriften als Thema, Informationsverbreitung, Serielle Veröffentlichungen, Wissenschaftliche Kommunikation, Medical journalism, Journals as topic, Information dissemination, Serial publications, Scholarly Communication

## Abstract

Im Jahr 1864 wurde die weltweit älteste Zeitschrift im Gebiet der späteren Hals-Nasen-Ohren-Heilkunde, das deutschsprachige *Archiv für Ohrenheilkunde* von den Herausgebern Anton von Tröltsch (Würzburg), Adam Politzer (Wien) und Hermann Schwartze (Halle/S.) gegründet. Zuvor waren in medizinischen Universalzeitschriften auch schon HNO-Themen veröffentlicht worden. Es folgte in den nächsten Jahrzehnten die Gründungsphase zahlreicher Zeitschriften im Gebiet der späteren Hals-Nasen-Ohren-Heilkunde, deren wechselvolle Geschichte bis in die Gegenwart dargestellt wird. Besonderes Augenmerk wird dabei auf den historischen und persönlichen Kontext der Herausgeber neu gegründeter Zeitschriften sowie deren Verlage und Verleger gelegt. Die durch Zukäufe und Fusionen von Verlagen sich verändernde Zeitschriftenlandschaft wird ausführlich beschrieben. Den größten Einfluss auf die Zeitschriftentitel und deren Inhalte hatte dabei die in Deutschland bis in die 1920er-Jahre dauernde Vereinigung des Fachs aus der Otologie und der Laryngorhinologie. Es wird der Versuch unternommen, die wichtigsten Titel in ihrem historischen Verlauf darzustellen. Erwähnt werden alle wichtigen Herausgeber der deutschsprachigen HNO-Zeitschriften, wobei es nicht möglich war, die immer zahlreicher werdenden Namen der Herausgeber der aktuellen Zeitschriften aufzunehmen. Ein Kapitel beschäftigt sich ausschließlich mit der Entwicklung der Zeitschriftenverlage. Die eingefügten Tabellen und Abbildungen sollen helfen, die Verwirrung durch immer wieder ähnlich lautende Namensgebungen der Zeitschriften durch Darstellung des historischen Verlaufs der Journale aufzulösen.

## Abstract (en)

In 1864, the worldwide oldest journal in an area of the later established specialty of otorhinolaryngology was founded as the German *Archiv für Ohrenheilkunde* (“Archive of Otology”) by its first editors Anton von Tröltsch (Würzburg), Adam Politzer (Vienna), and Hermann Schwartze (Halle/S.). Ear, nose, and throat (ENT) topics had previously been published in universal medical journals. In the next few decades, numerous journals in the field of ENT were founded, the eventful history of which is presented up to the present day. Particular attention is paid to the historical and personal context of the editors of newly founded magazines and their publishers. The journal landscape, which was changing through acquisitions and mergers of publishers, is described in detail. The merging of the specialties of otology and laryngo-rhinology in Germany, which lasted until the 1920s, had a profound influence on journal titles and contents. An attempt is made to present the most important titles in their historical development. All the important editors of the German ENT journals are mentioned, although it was not possible to include the names of the editors of the current journals, which are becoming more and more numerous. One chapter deals exclusively with the development of journal publishers. The inserted tables and figures will help to resolve some of the confusion caused by repeated similar names of journals by showing their historical development.

Johann Just von Berger (1723–1791) aus Celle, seit 1774 königlicher Leibarzt von Christian VII., König von Dänemark und Norwegen, litt in seinen letzten Lebensjahren an einer schweren Ohrerkrankung mit Schwindelanfällen, fortschreitender Taubheit und quälenden Ohrgeräuschen. Auch schon vor 250 Jahren waren die Therapieoptionen von Innenohrerkrankungen nur begrenzt. Operationen galten als Heilversuche in verzweifelten Fällen. Der Leidensdruck des ärztlichen Patienten war vermutlich enorm. Nach einer Trepanation des Mastoids und nachfolgenden Wundspülungen starb der Patient 1791 in Kopenhagen an einer eitrigen Meningitis [8]. Es war der erste bis dahin bekannt gewordene Todesfall nach Warzenfortsatzeröffnung. Wegen der herausragenden Stellung des Patienten am Dänischen Hof wurde der Fall schnell bekannt. Dies führte bis Mitte des 19. Jahrhunderts zur Ächtung jeglicher Mastoidchirurgie als Kunstfehler. Durch eine Erwähnung in den Betrachtungen „Über die Eröffnung des Warzenfortsatzes“ durch Hermann Schwartze (1837–1910), der Erstveröffentlichung zur wissenschaftlich begründeten Mastoidektomie 1873, erlangte der tragische Fall erneute Bedeutung. Baron Dr. von Berger galt lange Jahre als Märtyrer der Ohrchirurgie [83]. Schwartze, ein Pionier der Ohrchirurgie aus Halle, war Mitherausgeber und einer der Gründer des *Archivs für Ohrenheilkunde*, der weltweit ältesten, ausschließlich einem Gebiet der späteren Hals-Nasen-Ohren-Heilkunde gewidmeten und bis heute als *European Archives of Oto-Rhino-Laryngology and Head & Neck* existierenden Zeitschrift.

Der Austausch der Ärzte untereinander über Krankheiten, neue Therapien und medizinische Erfahrungen erfolgte bis Anfang des 19. Jahrhunderts ausschließlich über Briefkontakte, persönliche Besuche oder über Lehrbücher. Erst mit der Herausbildung der Medizin als Naturwissenschaft wurde es notwendig, andere Formen des Wissensaustausches zu finden, um mit der sich schnell entwickelnden Medizin als Wissenschaft Schritt zu halten. Im Jahr 1670 wurde weltweit das erste Fachjournal für Medizin als *Miscellanea curiosa medico-physica Academiae Naturae Curiosorum sive Ephemeridum medico-physicarum germanicarum curiosarum* von den Mitgliedern der 1652 gegründeten Leopoldina (Academia Naturae Curiosorum) ins Leben gerufen. Rudolf Virchow (1821–1902) beschrieb 1865 die Bedeutung dieser ersten Gründung eines medizinischen Periodikums wie folgt: „Von seiner Gründung in den letzten Decennien des 17. Jahrhunderts an hat es als ein Vorbild gedient, zunächst für andere Gesellschaftsschriften, sodann, namentlich seit dem Ende des vorigen Jahrhunderts, für immer zahlreichere, theils universelle, theils specialistische Fachjournale“ [100]. Schon in der ersten Ausgabe des ersten Bands der *Miscellanea curiosa* waren HNO-Themen vertreten. Damals noch in der üblichen Sprache der Gebildeten, Latein, schreibt Philippi Jacobi Sachs (1627–1672) über mögliche Zusammenhänge des Ohrs mit anderen Körperregionen [71]. Noch 175 Jahre später, 1845, wurde dieser Text in dem praktischen Handbuch der Ohrenheilkunde von Martell Frank (1810–1889) zitiert [30].

Heute, 350 Jahre später, ist der Wissenschaftsaustausch weitgehend digitalisiert und internetbasiert. Die wissenschaftlichen Fachzeitschriften mit ihren Peer-Review-Verfahren repräsentieren aber nach wie vor die „Container“, also die äußere Form des wissenschaftlichen Austausches. Daneben sind wissenschaftliche Kongresse, praktisch-wissenschaftliche Kurse und Klinikbesuche im Wissenschaftsaustausch und in der Verbreitung neuer Methoden nach wie vor fest etabliert. Mit der vorgelegten Darstellung soll die historische Entwicklung der HNO-Heilkunde in Deutschland aus der Perspektive der deutschsprachigen wissenschaftlichen HNO-Zeitschriften beleuchtet werden.

## Gründungsphase und Entwicklung im 19. Jahrhundert

Neben den *Miscellanea curiosa* gab es Ende des 18. Jahrhunderts eine Reihe von Gründungen medizinischer Zeitschriften, welche sich erst allmählich einer wissenschaftlichen Grundlage der Medizin bedienten. Christoph Wilhelm Hufeland (1762–1836), der Leibarzt Friedrich Wilhelms III., König von Preußen, gründete 1795 eine der zu dieser Zeit am weitesten verbreiteten Zeitschriften, das *Journal der praktischen Arzneikunde und Wundarzneikunst*, welches noch den verschiedensten Richtungen der Medizin, von der Homöopathie bis zum tierischen Magnetismus, Platz einräumte [5]. Das Journal wurde bis zu Hufelands Tod in 83 Bänden herausgegeben und gehörte zu den angesehensten, reichhaltigsten und lehrreichsten medizinischen Zeitschriften in deutscher Sprache. Darüber hinaus bescherte die erfolgreiche Herausgabe der Zeitschrift Hufeland einigen Wohlstand: „Außer dem wissenschaftlichen Nutzen, den das für die Aufrechterhaltung der erfahrungsmäßigen Medicin (im Gegensatz zur hypothetischen) bestimmte ‚Journal der praktischen Heilkunde‘ stiftete, wurde es auch für H. eine gute Stütze in der Noth, eine Hauptquelle seines Vermögens, indem er sich zum Grundsatz machte, die Einkünfte davon nicht auszugeben, sondern zurückzulegen. … Durch seine litterarischen Arbeiten, besonders die Makrobiotik und das Journal hatte er so viel gewonnen, daß er ein Capital von 10.000 Thlrn. besaß …“ [34].

Mit der Gründung des Deutschen Kaiserreichs 1871 unter preußischer Führung entstand in Deutschland ein Nationalstaat mit einheitlichem Markt und einheitlichen Regeln. Der liberale Charakter der entstehenden Zivilgesellschaft beförderte Bildung und Wissenschaft v. a. in der Reichshauptstadt Berlin. Es ist deshalb nicht verwunderlich, dass in dieser Zeit wesentliche Impulse der medizinischen Entwicklungen von Berlin und insbesondere von der Charité ausgingen. Die Revolution von 1848/49 hatte den Grundstein für eine demokratische Entwicklung in Deutschland gelegt, auch wenn mit deren Scheitern zunächst die Entwicklung des politischen Bürgertums mit seinem freiheitlichen Denken und seinen Vorstellungen von freiheitlicher Wirtschaft zurückgeworfen wurde. Die einsetzende Industrialisierung und die rasante wirtschaftliche Entwicklung im letzten Drittel des 19. Jahrhunderts brachte eine schnelle Ausweitung der Verkehrsinfrastruktur. In die Reichshauptstadt Berlin wanderten unzählige Neubürger als Arbeitskräfte für die sich schnell vergrößernden Industriebetriebe ein. Diese industrielle Revolution führte somit zu einer Konzentration der Bevölkerung in den Großstädten mit der Folge einer nie dagewesenen Notwendigkeit der medizinischen Versorgung auf engem Raum. Die vom Reichskanzler Bismarck in dieser Zeit angestoßene Sozialgesetzgebung mit der Einführung einer Kranken- und Unfallversicherungspflicht für Arbeiter (1883/84) sowie die Einführung einer Rentenversicherung 1891 führten zu einer Verbesserung der sozialen Lage der Arbeiterschaft (vgl. Draheim 2014 [26]).

Mit der Zunahme der Anzahl der Studierenden der Medizin und dem zunehmenden Wohlstand der Ärzte in Deutschland bildete sich in der zweiten Hälfte des 19. Jahrhunderts sehr schnell ein eigenes medizinisches Verlagswesen heraus. Im Jahr 1878 erschienen 425 medizinische Titel und 67 Zeitschriften in 150 Firmen. Parallel zur zunehmenden Spezialisierung der Medizin erfolgte auch eine Spezialisierung des medizinischen Verlagswesens. Diese Spezialisierung fand ihren Höhepunkt in der erfolgreichen Gründung der ersten ausschließlich medizinischen Verlage durch Georg Thieme (1860–1925) 1886 in Leipzig und Samuel Karger (1863–1935) 1890 in Berlin. Durch den Verlagswechsel zahlreicher Programmbereiche und Titel kam es zudem zu einer Spezialisierung von Verlagen mit Schwerpunkt Medizin. So entwickelten zahlreiche Verlage wie F. C. W. Vogel, Leipzig, J. F. Bergmann, Wiesbaden, Gustav Fischer, Jena, J. F. Lehmanns, München, Urban & Schwarzenberg, Wien, Berlin, nach 1904 auch Springer, Berlin, eigenständige Verlagsprogramme in der Medizin mit Zeitschriften und Büchern (vgl. Jäger, S. 473–483 [40]). Neben universellen Zeitschriften wurden vermehrt spezialisierte Zeitschriften einzelner Fachgebiete publiziert. Meist wurden in Letzteren Originalarbeiten veröffentlicht, anfangs als Falldarstellungen und Beschreibungen von neuen Behandlungsmethoden, später dann als klinische Studien. In dieser Zeit waren auch Fallstatistiken aus einzelnen Kliniken beliebt, welche einen guten Überblick über die klinische Umsetzung neuer Therapien und Operationsmethoden boten.

Mit der Herausbildung der medizinischen Fachdisziplinen Ende des 19. Jahrhunderts war nicht nur die Gründung spezieller klinischer Ambulanzen und neuer Krankenhausabteilungen verbunden, sondern es begann auch die Abgrenzung der Fächer untereinander. Die heute in Deutschland in der Weiterbildungsordnung fixierten Fachdefinitionen begannen sich erst herauszubilden. „Die dynamische Entwicklung unseres Fachgebietes in mehr als 100 Jahren spiegelt die stetige Suche nach dessen Stellenwert im Zusammenspiel mit den benachbarten Fachgebieten wider“ [92]. Zahlreiche Zeitschriftenneugründungen in dieser Zeit führten deshalb den Zusatz „… und ihre Grenzgebiete“. Wegen der zunehmenden Zahl von Zeitschriften im gleichen Fachgebiet war es für den in der Praxis tätigen Arzt nicht mehr möglich, einen aktuellen Überblick über alle neuen Entwicklungen in seinem Fachgebiet zu behalten. Es entstanden die meist als „Zentralblatt“ bezeichneten Referate-Blätter. Besonders der Springer-Verlag platzierte Anfang des 20. Jahrhunderts ein ganzes System medizinischer Referateorgane, die ausschließlich medizinische Referate, Kongresspublikationen und Rezensionen veröffentlichten (vgl. Sarkowski, S. 180 [73]). Bereits 1884 war im Verlag Hirschwald, Berlin, das *Internationale Zentralblatt für Laryngologie, Rhinologie und verwandte Wissenschaften* etabliert worden. Dieses Journal wurde später als *Zentralblatt Hals-Nasen-Ohrenheilkunde, plastische Chirurgie an Kopf und Hals, Organ der Deutschen Gesellschaft für Hals-Nasen-Ohrenheilkunde, Kopf- und Halschirurgie* als Kongress-Publikationsorgan fortgeführt und 1996 vom Springer-Verlag eingestellt.

Die klassische Universalzeitschrift ist die bis heute existierende *Deutsche Medizinische Wochenschrift* (*DMW*), gegründet 1875 von Paul Börner (1829–1885) im Verlag Georg Reimer, ab 1887 im Thieme-Verlag. Sie war in dieser Zeit wegen der Themen Medizinalreform, Hygiene und Zellularpathologie von Rudolf Virchow beeinflusst und galt wegen der Veröffentlichungen zu den Themen Cholera, Diphtherie und Tetanus als Hausblatt von Robert Koch (1843–1910) und Emil von Behring (1854–1917) [89]. Ein weiteres bis heute publiziertes, universelles Journal war das 1847 von Rudolf Virchow und Benno Reinhardt (1819–1852) gegründete* Archiv für pathologische Anatomie und Physiologie und klinische Medizin (Virchows Archiv)*. Üblicherweise lagen Verlagsübernahmen ausschließlich wirtschaftliche Motive der Verlage zugrunde. Im Fall von Virchows Archiv erfolgte aber auf Drängen der Herausgeber die Übernahme im Jahr 1920 durch den Springer-Verlag. Die Zeitschrift war in den Jahren davor beim Verlag Georg Reimer (später Verlag Walter de Gruyter) in Berlin mit rückläufigen Zahlen nur von einem „kleinlichen Prokuristen“ betreut worden (vgl. Sarkowski, S. 252 [73]). Insgesamt kam es in der ersten Hälfte des 20. Jahrhunderts zu zahlreichen Verlagsübernahmen und damit zu Fusionen von Zeitschriften sowie zu einer Konsolidierung des Markts medizinwissenschaftlicher Zeitschriften.

Eine besondere Entwicklung nahmen um die vorletzte Jahrhundertwende die Verbreitung und der Gebrauch der deutschen Sprache als Wissenschaftssprache. Anfangs war Deutsch in der Wissenschaft noch als echte Lingua franca weit verbreitet, d. h. der Gebrauch von Deutsch war als Kommunikationsform zwischen Nichtmuttersprachlern verschiedener Sprachen üblich. So diente Deutsch Ende des 19. Jahrhunderts häufig als Sprache internationaler Kongresse und fungierte als Übersetzungssprache von internationalen Referateorganen. Wissenschaftler aus Skandinavien oder den Niederlanden publizierten z. B. selbstverständlich in Deutsch, und in Osteuropa oder selbst in Russland erschienen deutschsprachige wissenschaftliche Zeitschriften. Deutsch als internationale Wissenschaftssprache war zu dieser Zeit dem Englischen ebenbürtig. Erst durch die Folgen der beiden Weltkriege verlor Deutsch als internationale Wissenschaftssprache an Bedeutung. Durch den ökonomischen Ruin der deutschsprachigen Länder und durch den Verlust großer Teile der habsburgischen Länder Osteuropas, in denen Deutsch als Amtssprache galt, wurde Deutsch immer weniger als Lingua franca benutzt. Die Politik der Vertreibung und Ermordung von jüdischen Wissenschaftlern und Intellektuellen durch das Naziregime führte zudem zu einem dramatischen Verlust an Wissenschaftspotenzial in Deutschland (vgl. Ammon 2001 [2]).

## Zeitschriftenchroniken und die Rolle exzellenter Wissenschaftler und der Verlage bei der Gründung neuer Zeitschriften

Mit der Entwicklung der Medizin als Naturwissenschaft am Beginn des 19. Jahrhunderts nahm das Bedürfnis nach wissenschaftlichem Austausch zu, und es entstand ein zunehmend wirtschaftlicher Markt für Publikationen. Die Zahl der Ärzte und der Medizinstudenten im Deutschen Reich stieg in dieser Zeit deutlich. Dies führte einerseits zu Neugründungen von zahlreichen Zeitschriften durch herausragende Wissenschaftler. Andererseits geriet das Feld der medizinisch-wissenschaftlichen Publikationen zunehmend in den Blick von Verlagen, welche zahlreiche Zeitschriften unterschiedlichster Aufmachung, Erscheinungsintervalle, Inhalte, Rubriken und Zielgruppen gründeten. Nicht alle Zeitschriften konnten sich am Markt behaupten und wurden wiedereingestellt, von anderen Verlagen übernommen oder mit anderen Zeitschriften fusioniert. Einzelne Journale und Verlage existieren jedoch bis heute und haben nach wie vor eine herausragende nationale und auch internationale Bedeutung erlangt.

Betrachtet man die heutigen deutschsprachigen und von deutschen Verlagen herausgegebenen HNO-Zeitschriften, so gehen die meisten dieser Journale auf Gründungen in der zweiten Hälfte des 19. und der ersten Hälfte des 20. Jahrhunderts zurück. Das Verhältnis von Verlagen, Herausgebern und wissenschaftlichen Fachgesellschaften unterliegt bis heute einem steten Wandel. Die wissenschaftliche Bedeutung und die Funktion als Publikationsorgan von wissenschaftlichen Fachgesellschaften in Deutschland, Österreich und der Schweiz hatten von jeher großen Einfluss auf den wirtschaftlichen Erfolg oder Misserfolg der Zeitschriften. Dies konnte zur Einstellung, Fusion oder zur Jahrzehnte dauernden Publikation einer einzelnen Zeitschrift führen. Noch heute wird dies sichtbar an der bis zu dreistelligen Nummer der Zeitschriftenbände (Jahrgänge). So trägt das Volume des aktuellen Bands von 2020 der *European Archives of Oto-Rhino-Laryngology and Head & Neck* eine dreistellige Nummer. Die Zeitschrift wurde 1864 als weltweit älteste, ausschließlich einem Gebiet der späteren Hals-Nasen-Ohren-Heilkunde gewidmete Zeitschrift, dem *Archiv für Ohrenheilkunde* gegründet.

Titeländerungen, Einstellungen und Fusionen der deutschsprachigen wissenschaftlichen HNO-Zeitschriften führen auf den ersten Blick zu einer gewissen Unübersichtlichkeit in ihrer geschichtlichen Betrachtung. Es wird in diesem Kapitel der Versuch unternommen, die komplexe historische Entwicklung der deutschsprachigen HNO-Zeitschriften möglichst übersichtlich darzustellen. Die Recherche zur Zeitschriftenentwicklung stützte sich v. a. auf im Web frei zugängliche Bibliotheksdatenbanken wie WorldCat (www.worldcat.org), HathiTrust (www.hathitrust.org) oder die Zeitschriftendatenbank ZDB der Deutschen Nationalbibliothek und der Staatsbibliothek Berlin (www.zdb-katalog.de). Es ist bekannt, dass jede Literaturrecherche in Abhängigkeit vom eingesetzten Aufwand und der Möglichkeit zu einem freien Online- oder traditionellen Zugang zu Bibliotheken einer Färbung unterliegt. So kann für die Erstellung von Leitlinien eine kostenintensive Literaturrecherche notwendig sein. Andererseits führen auch der Kontext des Autors und die Zielsetzung der historischen Betrachtung unweigerlich zu einem Bias. Die Autoren nehmen deshalb nicht für sich in Anspruch, ein perfektes und vollständiges Bild der deutschsprachigen wissenschaftlichen HNO-Zeitschriften abzubilden. Vielmehr handelt es sich um eine eher kursorische historische Betrachtung aus Sicht des Klinikers im Jahr 2020.

Zur besseren Übersicht sind die Zeitschriftentitel in den Tabellen ohne eindeutigen Zeitbezug, nur in der Reihenfolge ihrer Erwähnung, durchnummeriert. Dies soll helfen, bei den immer wieder ähnlichen Namensgebungen der Zeitschriftentitel eine gewisse Übersicht zu behalten. Es hat sich bei der Recherche herausgestellt, dass bei Fusionen und Übernahmen von Zeitschriftentiteln die Nummerierung der Bände eine wichtige Orientierung darstellt. Die Zeitschriftenbände (Volumes) sind deshalb fett dargestellt. Über die Bedeutung der Zeitschriftentitel und die Titeländerungen kann im Nachhinein nur eine grobe Einschätzung zum Inhalt und zum Ziel derselben gegeben werden. Während die ursprünglichen Zeitschriftengründungen vor der Fusionierung des Fachgebiets noch die einzelnen Organsysteme, wie Ohr, Nase oder Kehlkopf im Fokus hatten, sollten die Zusätze „Archiv“, „Zentralblatt“ oder „Zeitschrift“ Hinweise auf den Schwerpunkt Originalarbeiten, Referate- und Buch- oder Kongressbesprechungen oder eine Mischung von allem anzeigen. Im Gegensatz hierzu bezieht sich die Zeitschrift der American Laryngological, Rhinological and Otological Society, Inc. mit dem Titel *The Laryngoscope* zwar nur auf ein Organgebiet unseres Fachs. Darüber hinaus soll die Bedeutung der Spiegeluntersuchung als Grundlage für die Bezeichnung der 1896 gegründeten Zeitschrift gedient haben: „So, the journal is dedicated to serve as an illuminating instrument in the continuing examination of the diseases we treat in our specialty“ [6].

Medizinisch-wissenschaftliche Verlage waren seit ihrer Gründung stets bestrebt, geeignete Wissenschaftler und Ärzte zu finden, die durch ihre Persönlichkeit und Stellung im jeweiligen Fachgebiet als Herausgeber die Zielsetzung und den Charakter einer Zeitschrift bestimmen und geeignete Autoren verpflichten konnten. Daneben bestand das Interesse der Verlage darin, durch die Gewinnung in ihrer Zeit schon berühmter Mediziner für Buchprojekte und Zeitschriften-Herausgeberschaften das Ansehen des Wissenschaftlers auf den Verlag ausstrahlen zu lassen und damit zum wirtschaftlichen Erfolg beizutragen. Während der Verleger die unternehmerische Leistung erbringt und das wirtschaftliche Risiko trägt, besteht die Tätigkeit eines Herausgebers im Wesentlichen aus dem Prüfen und Zusammenstellen von Beiträgen zu Sammlungen (z. B. Lexika, Zeitschriften, Schriftreihen) [58]. Die Herausgeber agieren nicht nur als Botschafter einer Zeitschrift, sondern verantworten auch die Qualität des Inhalts einer Zeitschrift und tragen somit wiederum zu deren wirtschaftlichen Erfolg bei. Bei der Literaturrecherche in den zugänglichen Verlagsquellen findet man eine Vielzahl von Zeitschriftengründungen, -umbenennungen und -zusammenlegungen [11, 32, 33, 45, 47, 73, 86, 89]. Während die Neugründungen und Zusammenlegungen i. d. R. unternehmerische Gründe haben, gibt es für die Umbenennungen häufig inhaltliche Gründe. Dies ist u. a. mit der Entstehung der Hals-Nasen-Ohren-Heilkunde als gemeinsames Fach von Ende des 19. bis Anfang des 20. Jahrhunderts der Fall. So erfolgte 1915 z. B. die Umbenennung des *Archivs für Ohrenheilkunde*, Vorgänger der aktuellen *European Archives of Oto-Rhino-Laryngology and Head & Neck*, in *Archiv für Ohren‑, Nasen- und Kehlkopfheilkunde* in der Phase der Fusionen der Ohrenkliniken mit den Kliniken für Laryngologie und Rhinologie an deutschen Universitätskliniken und der später vereinbarten Körperschaft als Publikationsorgan der 1921 gegründeten Gesellschaft Deutscher Hals‑, Nasen- und Ohrenärzte.

Da die Herausgeberschaft der einzelnen Zeitschriften häufiger wechselt als die einzelnen Schritte der Titelhistorie, ist eine Darstellung der Herausgeberwechsel in den Bibliotheksdatenbanken nicht abgebildet. Zudem ist die Nennung des Herausgebers im Impressum auch heute nicht zwingend (vgl. Dernbach 2018 [13]). Bei der Recherche zu der vorgelegten Arbeit erwies es sich als schwierig, die Herausgeber der wechselnden Jahrgänge der Zeitschriften zu identifizieren, da die Namen nicht in den Bibliotheksdatenbanken erfasst sind. Bei der Zeitschriftendatenbank für Deutschland und Österreich (ZDB) wird lediglich die „herausgebende Körperschaft“, also z. B. die Fachgesellschaft erfasst. Die persönlichen Bedingungen und Beziehungen der Herausgeber als Persönlichkeiten sollten aber gerade den Reiz der vorgelegten Arbeit ausmachen. Es war also nötig, für eine möglichst vollständige Dokumentation der Herausgeber und ihrer Wechsel jeden einzelnen Band in die Hand zu nehmen und die in den Printversionen erfassten Herausgebernamen zu erfassen. Vornamen waren häufig nicht vollständig und Lebensdaten nie vorhanden und mussten zusätzlich recherchiert werden.

Der Begriff des Herausgebers ist sowohl publizistisch als auch rechtlich nicht eindeutig definiert, im Verlagswesen von heute ist der Herausgeber Bindeglied zwischen Verleger und Redaktion [13]. So wird häufig ein Herausgeberbeirat (Editorial Board), bestehend aus angesehenen Persönlichkeiten, aufgeführt, und die gestalterischen Aufgaben werden von nur einem oder wenigen Mitgliedern aus diesem Kreis, z. B. als Schriftleiter oder geschäftsführender Herausgeber (Editor in Chief), wahrgenommen. Hinzu kommen bei einigen Zeitschriften noch Rubriken- oder Sektionsherausgeber sowie manchmal auch ein eigentlicher (wissenschaftlicher) Beirat. In der heutigen Verlagsarbeit legt die Herausgebersitzung, zusammen mit der wissenschaftlichen Fachgesellschaft als Organschaft, i. d. R. die fachliche und strategische Ausrichtung der Zeitschrift fest. Personalentscheidungen in Bezug auf die Mitgliedschaft im Herausgebergremium werden ebenfalls in der Herausgebersitzung getroffen. Der Verlag hat allerdings immer ein Mitspracherecht, versteht sich aber i. Allg. als Dienstleister für die Herausgeber. Mittlerweile besteht ein Trend zum Rückgang der Printabonnements, sodass v. a. einige englischsprachige Zeitschriften ausschließlich online publiziert werden. Ein besonderes Problem von einigen deutschsprachigen medizinwissenschaftlichen Zeitschriften ist die internationale Wahrnehmbarkeit durch die fehlende Listung in der Medline-Datenbank bzw. im Social Sciences Citation Index (SSCI). In diesen von US-amerikanischen Institutionen und Firmen dominierten Datenbanken sind kaum nichtenglischsprachige Publikationen gelistet, sodass ein Bias in der Forschung bestimmter Gebiete anzunehmen ist.

Ein Problem stellt die seit den 1990er-Jahren begonnene sog. Zeitschriftenkrise dar: Aufgrund des Konzentrationsprozesses auf dem Markt der Wissenschaftsverlage kam es seit dieser Zeit zu deutlichen Preissteigerungen für Zeitschriften und damit zu einem erheblichen ökonomischen Problem für die wissenschaftlichen Bibliotheken. Kritisiert wurde die hohe Rendite der Wissenschaftsverlage bei der Verwertung von mit öffentlichen Mitteln geförderten Forschungsergebnissen. Es folgte der Boykott eines Teils der großen Wissenschaftsverlage durch eine ganze Reihe von Bibliotheksverbünden. In der Folge entstand die „Open-Access-Bewegung“, welche einen freien Zugang zu wissenschaftlicher Literatur einschließlich Primär- und Metadaten im Internet garantieren soll. In verschieden europäischen Ländern wurde die Gründung von Open-Access-Zeitschriften gefördert, so z. B. in Deutschland das Portal „German Medical Science“. Mittlerweile haben sich verschiedene Geschäftsmodelle etabliert. Über Publikationsgebühren soll sich der Autor an den Prozesskosten der Publikation beteiligen. In institutionellen Mitgliedschaftsmodellen zahlen Forschungsinstitutionen über eine Jahresgebühr die Veröffentlichung der Forschungsergebnisse in einem Open-Access-Journal. Open-Access-Publikationen werden häufiger gelesen, konnten bisher jedoch nicht die traditionellen Zeitschriften in den Impact-Faktor-generierenden Datenbanken verdrängen. Mittlerweile ist es zu einem problematischen Ökonomisierungsprozess im Open-Access-Betrieb gekommen bis hin zur Etablierung von „Predator-Journals“ mit fragwürdigen Geschäftspraktiken, aggressiver Werbung und mangelhaften Peer-Review-Prozessen (vgl. [103, 104]).

Einen neuen Weg geht das „DEAL-Projekt“ der Allianz der deutschen Wissenschaftsorganisationen, welches einerseits bundesweit durch neue Vertragsmodelle mit großen Wissenschaftsverlagen wie Springer Nature den Zugang zu elektronischen Zeitschriften erleichtert und andererseits es den Autorinnen und Autoren der beteiligten Wissenschaftseinrichtungen ermöglicht, in den Zeitschriften der beteiligten Verlage „Open Access“ zu publizieren. Für Springer Nature gilt dies für die HNO-Zeitschriften *HNO* und *European Archives of Oto-Rhino-Laryngology and Head & Neck* [57].

Zeitschriftenneugründungen, insbesondere wenn diese noch heute publiziert werden, sollen hier besonders herausgestellt werden. In chronologischer Reihenfolge sollen die Gründungsherausgeber und -verleger einiger Zeitschriftenneugründungen in ihrem historischen, wissenschaftlichen und persönlichen Kontext erwähnt werden. In der tabellarischen Zusammenstellung sind bei den Zeitschriften anfangs alle Herausgeber, später jedoch mit dem deutlichen Anstieg der Zahl der Herausgeber i. d. R. nur die Schriftleiter (Editor in Chief) aufgeführt, da diese auch historisch wesentlichen Einfluss auf die Gestaltung und Richtung der jeweiligen Zeitschrift hatten. Die historische Abfolge der Zeitschriften ist in den Tabellen dargestellt. Bei Änderungen des Titels der Zeitschriften oder bei einem Wechsel des Verlags durch Fusion oder Verkauf wird die Fortführung des Journals üblicherweise an der Nummerierung der Volumes sichtbar. Daneben wird jedoch auch bei Zeitschriftenneugründungen im Editorial Bezug auf die Vorgängerzeitschriften genommen, ohne dass die Volume-Nummern fortgeführt werden. Dem Leser im Jahr 2021 fällt es sicher leichter, von den derzeit publizierten HNO-Zeitschriften ausgehend einen Blick zurück zu den Zeitschriftengründungen zu werfen. Deshalb werden zunächst die aktuellen Publikationsorgane der deutschsprachigen wissenschaftlichen Fachgesellschaften in ihrer historischen Entwicklung dargestellt.

### Titelhistorie *European Archives of Oto-Rhino-Laryngology and Head & Neck*

#### *Official journal of the European Federation of Oto-Rhino-Laryngological Societies (EUFOS); official journal of the European Laryngological Society*, Berlin; Heidelberg: Springer [23]

Aus Anlass des 150-jährigen Jubiläums der Gründung des *Archiv für Ohrenheilkunde* (AfO) fand in der Nationalen Akademie der Wissenschaften Leopoldina in Halle/Saale vom 7.–10. Mai 2014 eine große Fachtagung mit Teilnehmern aus der ganzen Welt statt. Die *AfO* ist weltweit die älteste, ausschließlich einem Gebiet der späteren Hals-Nasen-Ohren-Heilkunde gewidmete Zeitschrift und existiert bis heute als *European Archives of Oto-Rhino-Laryngology and Head & Neck* [3, 65].

Wie in Tab. 1 (Zeitschrift Nr. 1, 7, 11 und 12) dargestellt, gehen die European Archives auf 3 Zeitschriftenneugründungen aus dem vorletzten Jahrhundert zurück: *Archiv für Ohrenheilkunde* (1864), *Archiv für Augen- und Ohrenheilkunde* (1869) und *Archiv für Laryngologie und Rhinologie* (1893). Die beiden Letzteren wurden 1922 zur *Zeitschrift für Hals‑, Nasen- und Ohrenheilkunde* (1922) vereinigt. Die European *Archives of Oto-Rhino-Laryngology and Head & Neck* zählen zu den ältesten HNO-Zeitschriften weltweit. Im Jahr 1864 wurde die Zeitschrift als* Archiv für Ohrenheilkunde* (*AfO*) in Würzburg im Verlag der Stahel’schen Buch- und Kunsthandlung gegründet. Folgt man der Zählung der Bände, lässt sich die Geschichte der Zeitschrift mit ihren Verlagswechseln oder Verlagsübernahmen (Übernahme eines ganzen Verlags inklusive aller Buch- und Zeitschriftentitel) gut nachverfolgen. Nach Verlagerung des Titels in den Leipziger Verlag F. C. W. Vogel im Jahr 1873 erfolgte 1915 die Umbenennung in *Archiv für Ohren‑, Nasen- und Kehlkopfheilkunde* und 1922 die Übernahme des Vogel-Verlags durch den Springer-Verlag. Nach diversen Änderungen des Titels der Zeitschrift erfolgt die Herausgabe noch heute mit Volume 277 im Springer-Verlag als *European Archives of Oto-Rhino-Laryngology and Head & Neck*.

**Table 1:** 

1	***Archiv für Ohrenheilkunde***
	Leipzig: Vogel, Würzburg: Stahel [1864–1870] **1**. 1864–**6**. 1873; N. F. 1 [= **7**]. 1873–**4** = **10**. 1875/76; **11**. 1876 – **97**. 1914/15, Forts.: Archiv für Ohren‑, Nasen- und Kehlkopfheilkunde
	Hrsg. (1864–1915): Adam Politzer, Wien, Hermann Schwartze, Halle, Anton von Tröltsch, Würzburg, Friedrich Kretschmann, Magdeburg, Paul Manasse, Straßburg
2	***Archiv für Ohren‑, Nasen- und Kehlkopfheilkunde: Organ d. Deutschen Gesellschaft der Hals‑, Nasen‑, Ohrenärzte***
	Berlin; Heidelberg; Göttingen: Springer **98**. 1915/16 – **154**. 1944/45, 3/4; **155** = 52. 1947/49;** 156**. 1949/50 – **185**. 1965, Forts.: Archiv für klinische und experimentelle Ohren‑, Nasen- und Kehlkopfheilkunde
	Hrsg. (1915–1945): Alfred Denker, Halle/Saale, Otto Voss, Frankfurt/M, Karl Wittmaack, Jena/Hamburg, Oskar Wagener, Göttingen, Johannes Zange, Jena, Alfred Güttich, Köln, Karl Beck, Heidelberg, Otto Mayer, Wien, Adolf Greifenstein, Königsberg, Siegfried Unterberger, Wien, Kaarlo Y. A. Meurman, Helsinki
	Hrsg. (1947–1965): Hermann Frenzel, Göttingen, Carl von Eicken, Berlin, Wilhelm Lange, Leipzig, Alfred Seiffert, Heidelberg, Otto Steurer, Hamburg, Johannes Zange, Jena, Gustav Hofer, Graz, Erhard Lüscher, Basel, Adolf Miehlke, Göttingen, Emil Schlander, Wien, Kaarlo Y. A. Meurman, Helsinki, Siegfried Unterberger, Klagenfurt, Max Schwarz, Tübingen, Fritz Zöllner, Freiburg/Br
3	***Archiv für klinische und experimentelle Ohren‑, Nasen- und Kehlkopfheilkunde***
	Berlin: Springer **186**. 1966 – **205**. 1973, Forts.: Archives of Oto-Rhino-Laryngology
	Hrsg. (1966–1973): Hermann Frenzel, Göttingen, Karl-Heinz Vosteen, Frankfurt/M, Fritz Zöllner, Freiburg/Br., Chlodwig Beck, Freiburg/Br., Heinrich Spoendlin, Zürich/Innsbruck, Konrad Fleischer, Hamburg/Gießen, Walter Messerklinger, Graz, Carl Rudolf Pfaltz, Basel, Max Schwarz, Tübingen, Franz Altmann, New York, Hans-Joachim Denecke, Heidelberg, Hans Engström, Uppsala/Göteborg, Paul Henry Holinger, Chicago, Adolf Miehlke, Göttingen, Masanori Morimoto, Kyoto, Jürgen Tonndorf, New York, Horst L. Wullstein, Würzburg, Walter Schätzle, Göttingen, Harold F. Schuknecht, Boston/M, Walter Kley, Mainz
4	***Archives of Oto-Rhino-Laryngology: Organ of the Deutsche Gesellschaft für Hals-Nasen-Ohren-Heilkunde, Kopf- u. Hals-Chirurgie*** *=* *Archiv für Ohren‑, Nasen- und Kehlkopfheilkunde*
	Berlin; Heidelberg [u. a.]: Springer **206**. 1974 – **246**. 1989, Forts.: European Archives of Oto-Rhino-Laryngology
	Hrsg. (1974–1989): Chlodwig Beck, Freiburg/Br., Heinrich Spoendlin, Zürich/Innsbruck, Fritz Zöllner, Freiburg/Br., John M. Frederickson, Toronto, Uwe Ganzer, Mannheim/Düsseldorf, Alan David Kornblut, Washington D. C.
5	***European Archives of Oto-Rhino-Laryngology: official journal of the European Federation of Oto-Rhino-Laryngological Societies (EUFOS)***
	Berlin; Heidelberg [u. a.]: Springer **247**. 1990 – **261**. 2004, 1
	Hrsg. (1990–2004): Chlodwig Beck, Freiburg/Br., Uwe Ganzer, Mannheim/Düsseldorf, Alan David Kornblut, Washington D. C., Heinrich Spoendlin, Zürich/Innsbruck, Gordon B. Snow, Amsterdam, Jan Olofsson, Bergen
6	***European Archives of Oto-Rhino-Laryngology and Head & Neck: official journal of the European Federation of Oto-Rhino-Laryngological Societies (EUFOS)***
	Berlin; Heidelberg [u. a.]: Springer **261**. 2004, 2 –
	Hrsg. (2004–): Uwe Ganzer, Düsseldorf, Gordon B. Snow, Amsterdam, Jochen Werner, Marburg, Jan Olofsson, Bergen, Oliver Sterkers, Paris, Manuel Bernal-Sprekelsen, Barcelona, Roland Laszig, Freiburg/Br., Marc Remacle, Luxembourg
**Weitere Vorgängertitel**	
7	***Archiv für Augen- und Ohrenheilkunde***
	Wiesbaden: Bergmann; Karlsruhe: Müller **1**. 1869/70 – **7**. 1878/79, Forts.: Zeitschrift für Ohrenheilkunde
	Hrsg. (1869–1879): Hermann Knapp, New York, Samuel Moos, Heidelberg, Ludwig Mauthner, Innsbruck/Wien
8	***Zeitschrift für Ohrenheilkunde***
	Wiesbaden: Bergmann **8**. 1879 – **34**. 1899, Forts.: Zeitschrift für Ohrenheilkunde mit besonderer Berücksichtigung der Rhinologie und der übrigen Grenzgebiete
	Hrsg. (1879–1899): Hermann Knapp, New York, Samuel Moos, Heidelberg, Otto Körner, Rostock, Arthur Hartmann, Berlin, Urban Pritchard, London
9	***Zeitschrift für Ohrenheilkunde mit besonderer Berücksichtigung der Rhinologie und der übrigen Grenzgebiete***
	Wiesbaden: Bergmann **35**. 1899 – **54**. 1907, Forts.: Zeitschrift für Ohrenheilkunde und für die Krankheiten der Luftwege
	Hrsg. (1899–1907): Hermann Knapp, New York, Otto Körner, Rostock, Arthur Hartmann, Berlin, Urban Pritchard, London
10	***Zeitschrift für Ohrenheilkunde und für die Krankheiten der Luftwege***
	Wiesbaden: Bergmann **55**. 1908 – **82**. 1922, Forts.: Zeitschrift für Hals‑, Nasen- und Ohrenheilkunde (12)
	Hrsg. (1908–1922): Hermann Knapp, New York, Otto Körner, Rostock, Arthur Hartmann, Berlin, Urban Pritchard, London, Gustav Killian, Freiburg/Br., Carl von Eicken, Berlin, Friedrich Siebenmann, Basel, Alfred Denker, Halle a. S.
11	***Archiv für Laryngologie und Rhinologie***
	Berlin: Hirschwald **1**. 1893/94 – **34**. 1921, Forts.: Zeitschrift für Hals‑, Nasen- und Ohrenheilkunde (12)
	Hrsg. (1893–1921): Bernhard Fränkel, Berlin, Georg Finder, Berlin, Ottokar Chiari, Wien, Paul Gerber, Königsberg, Otto Kahler, Freiburg/Br., Gustav Killian, Berlin, Hans Neumayer, München, Otto Seifert, Würzburg, Gustav Spiess, Frankfurt/M., Markus Hajek, Wien
12	***Zeitschrift für Hals‑, Nasen- und Ohrenheilkunde***/Gesellschaft Deutscher Hals‑, Nasen- und Ohrenärzte
	Berlin: Springer **1**. 1922 – **51**. 1944, aufgeg. in: Archiv für Ohren‑, Nasen- und Kehlkopfheilkunde (2)
	Hrsg. (1922–1944): Otto Körner, Rostock, Carl von Eicken, Berlin, Georg Finder, Berlin, Karl Wittmaack, Jena, Wilhelm Lange, Leipzig, Julius Hegener, Hamburg, Ernst Oppikofer, Basel, Reinhard Perwitzschky, Breslau, Erhard Lüscher, Basel, Bernhard Langenbeck, Bonn

**Figure 1:**
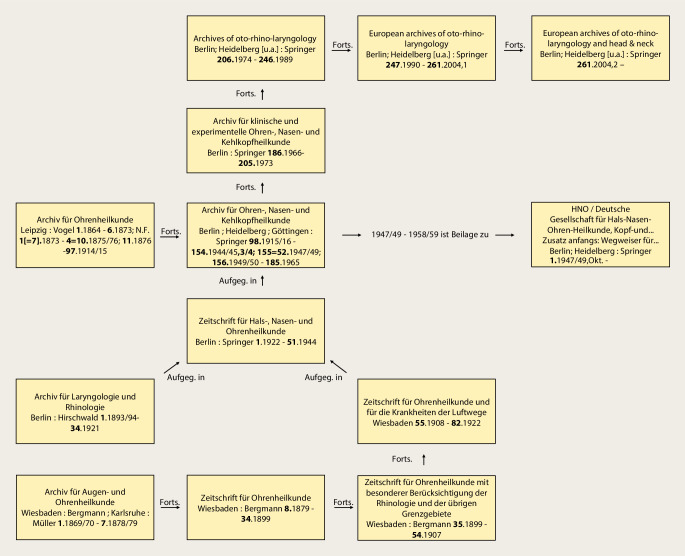

**Figure 2:**
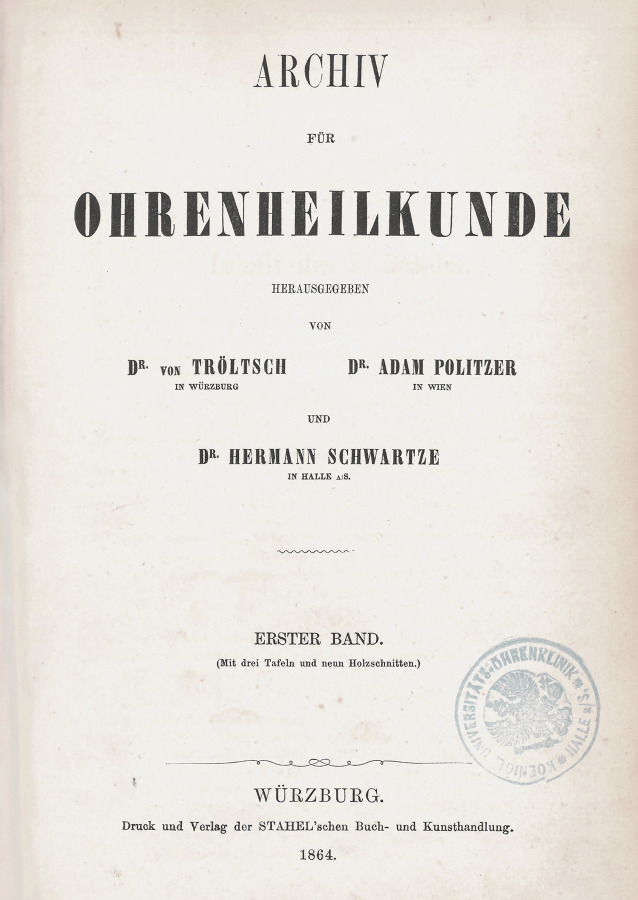

**Figure 3:**
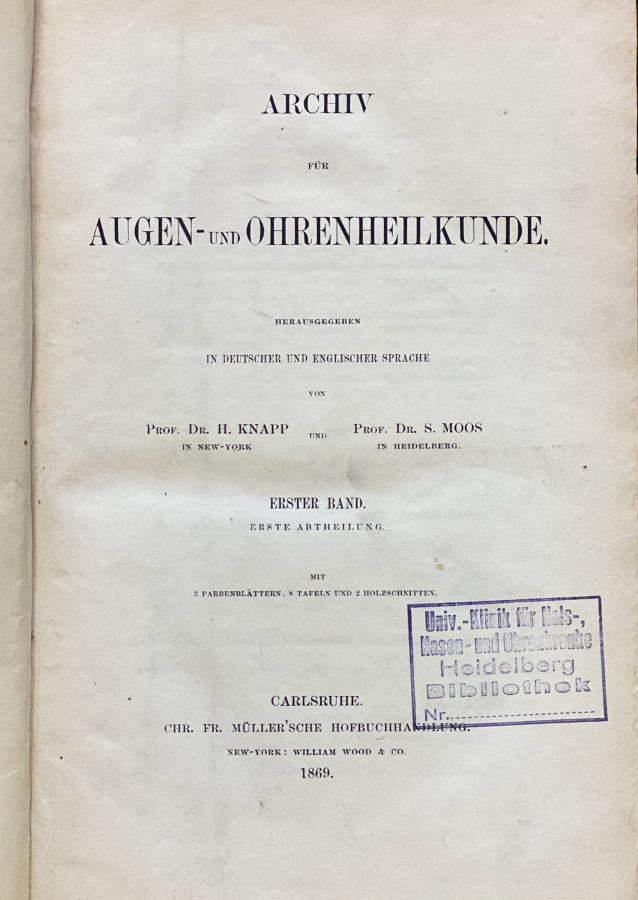

Im Verlauf der Jahre sind weitere Titel, hauptsächlich durch Kauf der Verlage Bergmann, Wiesbaden, und Hirschwald, Berlin, durch den Springer-Verlag im *AfO* aufgegangen. Auch dies lässt sich in der Tab. 1 anhand der Bandzählung gut nachvollziehen: Das 1869 gegründete *Archiv für Augen- und Ohrenheilkunde* (Bergmann) geht über diverse Titeländerungen und Abspaltungen 1922 durch den Verlagsverkauf an den Springer-Verlag. Ebenso kommt das 1893 bei Hirschwald in Berlin erstmals herausgegebene *Archiv für Laryngologie und Rhinologie *1922 durch Verlagsverkauf zu Springer. Beide Titel werden zur *Zeitschrift für Hals‑, Nasen- und Ohrenheilkunde* (*ZfHNO*) fusioniert. Die *ZfHNO* geht dann am Ende des 2. Weltkriegs im *Archiv für Ohren‑, Nasen- und Kehlkopfheilkunde* auf, deren Nachfolger heute wiederum die *European Archives* sind. Anschaulich ist in Abb. 1 der Verlauf der Titeländerungen und Fusionierungen in einem Flussdiagramm dargestellt. Die jeweiligen Titelblätter der neu gegründeten Vorgängerzeitschriften der *AfO* sind in den Abb. 2, 3, 4 und 5 abgebildet.

**Figure 4:**
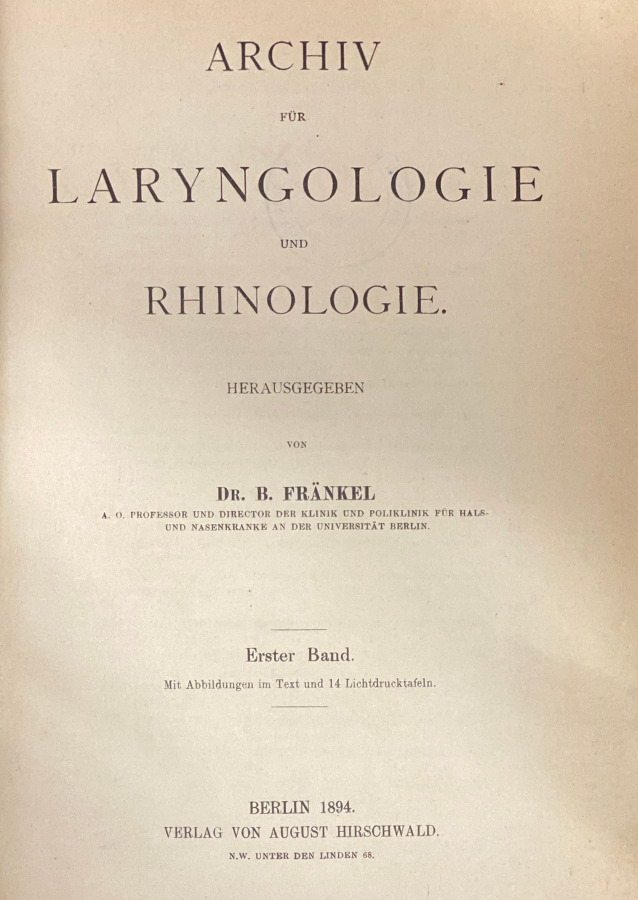

**Figure 5:**
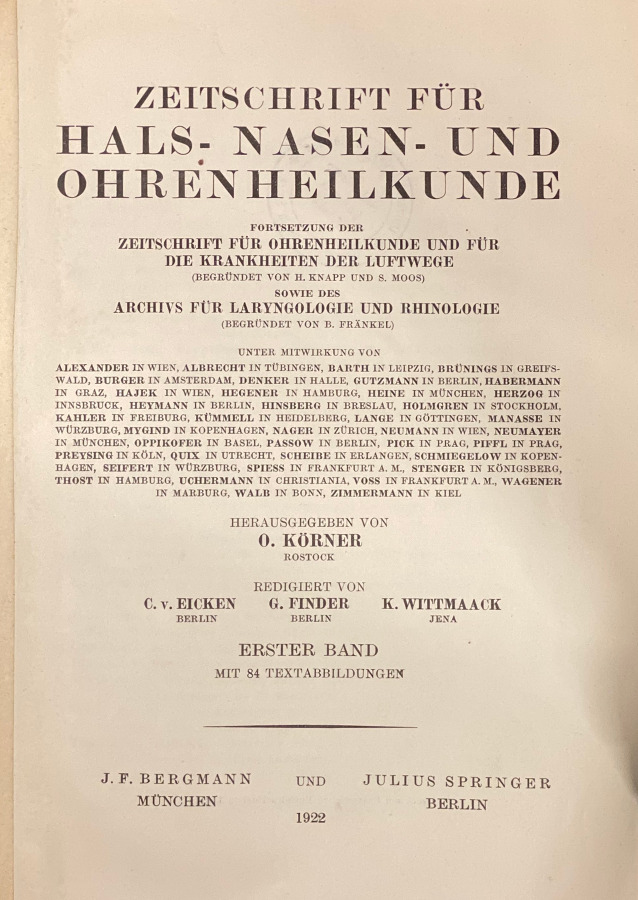

Im Verlauf des 19. Jahrhunderts sind die heute bekannten Verlage meist aus Buchhandlungen mit angeschlossener Druckerei hervorgegangen. So auch die seit 1753 bestehende Stahel’sche Buch- und Kunsthandlung in Würzburg. Für Anton von Tröltsch, im Jahr 1861 im Fach Ohrenheilkunde frisch habilitiert [81] und in dieser Zeit praktizierender Augen- und Ohrenarzt in Würzburg, und die beiden anderen Gründungsherausgeber, Adam Politzer und Herrmann Schwartze, lag es nahe, die Gründung des *Archiv für Ohrenheilkunde* einem lokalen „Verleger“ zu übertragen. Über die Gründung der Zeitschrift und die inhaltliche Struktur ist an anderer Stelle schon berichtet worden [62, 63]. Der „Gründungsmythos“ wird in gleicher Zeitschrift 1890 von Schwartze im Nachruf für Anton von Tröltsch und von Politzer zum 50. Jahrestag des Erscheinens 1914 wie folgt beschrieben: „Das Interesse, welches durch die ersten Publikationen von Tröltsch’s … für das bisher allgemein für unfruchtbar gehaltene Studium der Ohrenheilkunde erweckt war … führte zu der Idee der Begründung eines besonderen literarischen Organs, welches alles vereinigen sollte, was auf otologischem Gebiet gearbeitet wurde. Die erste Anregung ging (in einem Brief an von Tröltsch vom 28. März 1863) von einem Augenarzte, Dr. Zander in Chemnitz aus … Acht Monate später trat A. Politzer mit derselben Idee an von Tröltsch heran“ [66, 81]. Die ersten Herausgeber waren Dr. Anton von Tröltsch, aus Würzburg, Dr. Adam Politzer aus Wien und Dr. Hermann Schwartze aus Halle (Saale). Anfangs hatte von Tröltsch wegen zu erwartender ungenügender Zahl von Mitarbeitern und Abnehmern Bedenken an der Existenzfähigkeit der Zeitschrift. Erst durch die Zusage von Hermann Schwartze wurde die Zeitschriftengründung in Angriff genommen und später durch den Wechsel zum Verlag F. C. W. Vogel 1873 in Leipzig zum Erfolg geführt. Alle 3 Herausgeber waren bei der Gründung der Zeitschrift 1864 junge Männer (von Tröltsch 35, Politzer 29 und Schwartze 27 Jahre) mit großem Interesse an der Ohrenheilkunde und standen am Anfang ihrer ärztlichen und wissenschaftlichen Karriere. Im Nekrolog für Anton von Tröltsch beschreibt Hermann Schwartze 1890 die schwierige Anfangsphase des Archivs: „Die ersten Bände desselben folgten sich unregelmäßig und schleppend, und erst mit dem Übergang des Archivs in den Verlag von F. C. W. Vogel in Leipzig kam dasselbe in ein glattes Fahrwasser, als durch wachsende Zahl der Mitarbeiter die Beiträge reichlicher zuströmten und durch geregelten Geschäftsbetrieb des neuen Verlegers das schnellere Erscheinen der Arbeiten und die weitere Verbreitung des Archivs gesichert war“ (vgl. Schwartze 1890, Mudry 2015 [62, 81]). Es lässt sich auch heute noch nachvollziehen, dass die Gründer der Zeitschrift die weitere Verbreitung des *AfO* in einem großen Verlag im Blick hatten. Der Enkel des Verlagsgründers Carl Victor Lampe-Vischer hatte den F. C. W. Vogel-Verlag 1862 übernommen und das Verlagsprogramm in Richtung Medizin neu ausgerichtet (vgl. Sarkowski, S. 312 [73]). Leipzig war bis zum 2. Weltkrieg die führende Verlags- und Buchstadt Deutschlands. Mit dem Verkauf des Verlags F. C. W. Vogel 1930 an Springer gingen die *AfO* dann an diesen Verlag über, wo sie bis heute in kontinuierlicher Zählung der Zeitschriftenbände als *European Archives of Oto-Rhino-Laryngology and Head & Neck* unter der Schriftleitung von Manuel Bernal-Sprekelsen, Roland Laszig und Marc Remacle in englischer Sprache publiziert werden.

In ihrem Editorial zur ersten Nummer ihrer 1869 neu gegründeten Zeitschrift *Archiv für Augen- und Ohrenheilkunde* beziehen sich Hermann Knapp (1832–1911), New York, und Salomon Moos (1831–1895), Heidelberg, auf den bahnbrechenden Fortschritt, der mit der Erfindung des Augenspiegels für die Untersuchung von Auge und Ohr als Sinnesorgane eingeleitet worden war. Die „nahe Verwandtschaft der Augen- und Ohrenheilkunde … fordert dazu auf, beide vereinigt zu betreiben“ [51]. Knapp, 1859 bei Helmholtz in Heidelberg habilitiert, gründete 1862 hier eine Augenklinik für stationäre und ambulante Patienten. Wegen der Verzögerung beim geplanten Bau einer eigenen Universitätsaugenklinik in Heidelberg wanderte Knapp 1868 nach New York aus. Dem damaligen amerikanischen Trend entsprechend bezog Knapp bei der Gründung seines New York Ophthalmic and Aural Institute die Behandlung von Ohrenkrankheiten mit ein [54]. Zusammen mit Salomon Moos, seit 1866 außerordentlicher Professor für Ohrenheilkunde in Heidelberg, gründete Knapp 1869 die Zeitschrift *Archiv für Augen- und Ohrenheilkunde/Archives of Ophthalmology and Otology*, welche in New York bei William Wood in englischer Sprache und in Karlsruhe bei der Müller’schen Hofbuchhandlung in deutscher Sprache erschien. Diese Verbindung zwischen amerikanischer und deutscher Medizin hatte nicht nur die persönliche Beziehung zwischen Moos und Knapp in ihrer gemeinsamen Zeit in Heidelberg zur Ursache, sondern resultiert auch aus dem hohen Ansehen, welches die deutschen Spezialdisziplinen mit ihren soliden naturwissenschaftlichen Grundlagen zu dieser Zeit in den USA genoss (vgl. Koelbing 1979 [54]). Das Archiv wurde jeweils mit dem 8. Band 1879 als *Zeitschrift für Ohrenheilkunde* und als *Archiv für Augenheilkunde* beim Verlag Bergmann weitergeführt.

Bernhard Fränkel (1836–1911) gründete 1893 im Berliner Verlag August Hirschwald das *Archiv für Laryngologie und Rhinologie*. Dem Laryngologen Fränkel, Schüler von Rudolf Virchow, seit 1887 habilitiert, wurde 1887 die Leitung eines eigenständigen Instituts für Laryngologie und Rhinologie an der Berliner Charité übertragen. Er wurde 6 Jahre später zum Direktor der neugegründeten Klinik für Hals- und Nasenkranke berufen, welche er bis kurz vor seinem Tod 1911 leitete. Anschließend führte Georg Finder (1867–1931), Oberarzt an der Charité unter Fränkel und seinem Nachfolger Gustav Killian, die Zeitschrift bis zur Übernahme durch den Springer-Verlag 1921 als Herausgeber. Neben Finder waren noch die Laryngologen Ottokar Chiari (1853–1918), Wien, Paul Gerber (1863–1919), Königsberg, Gustav Killian (1869–1921), Berlin, Markus Hajek (1861–1941), Wien, Otto Kahler (1878–1946), Freiburg/Br., und Hans Neumayer (1865–1938), München, als Mitherausgeber tätig.

Die Neugründungen von Medizin-Journalen ging parallel einher mit der Gründung von medizinischen Fachgesellschaften. Allein zwischen 1850 und 1914 wurden 65 medizinische und naturwissenschaftliche Fachgesellschaften gegründet [68]. Im Jahr 1892 erfolgte die Gründung der Deutschen Otologischen Gesellschaft, 1894 die Gründung des Vereins Süddeutscher Laryngologen und 1905 der Deutschen Laryngologischen Gesellschaft. So war es logisch, dass auch die entsprechenden Zeitschriften zur gleichen Zeit gegründet wurden. Die Übernahme des Hirschwald-Verlags durch Springer im Jahr 1922 und die damit verbundene Verlagerung von Journalen hatte überwiegend familiäre oder unternehmerische Gründe. August Hirschwald hatte 1816 in Berlin eine Buchhandlung eröffnet und war ab 1826 verlegerisch tätig geworden. Schon früh war der Verlag August Hirschwald im Bereich Medizin sehr erfolgreich, hatte aber um die Jahrhundertwende mit den Verlagen F. C. W. Vogel, J. F. Bergmann, G. Fischer, S. Karger, G. Thieme und Urban & Schwarzenberg sehr starke Konkurrenz bekommen. Nach dem 1. Weltkrieg wurde der Hirschwald-Verlag von Springer übernommen [73]. So wurde das *Archiv für Laryngologie und Rhinologie* ab 1922 in der *Zeitschrift für Hals‑, Nasen- und Ohrenheilkunde* mit neuer Bandzählung fortgeführt.

### Titelhistorie *HNO*

#### *Deutsche Gesellschaft für Hals-Nasen-Ohren-Heilkunde, Kopf- und Hals-Chirurgie, Deutsche Akademie für Hals-Nasen-Ohren-Heilkunde, Kopf- und Hals-Chirurgie*, Heidelberg: Springer-Verlag [25]

**Figure 6:**
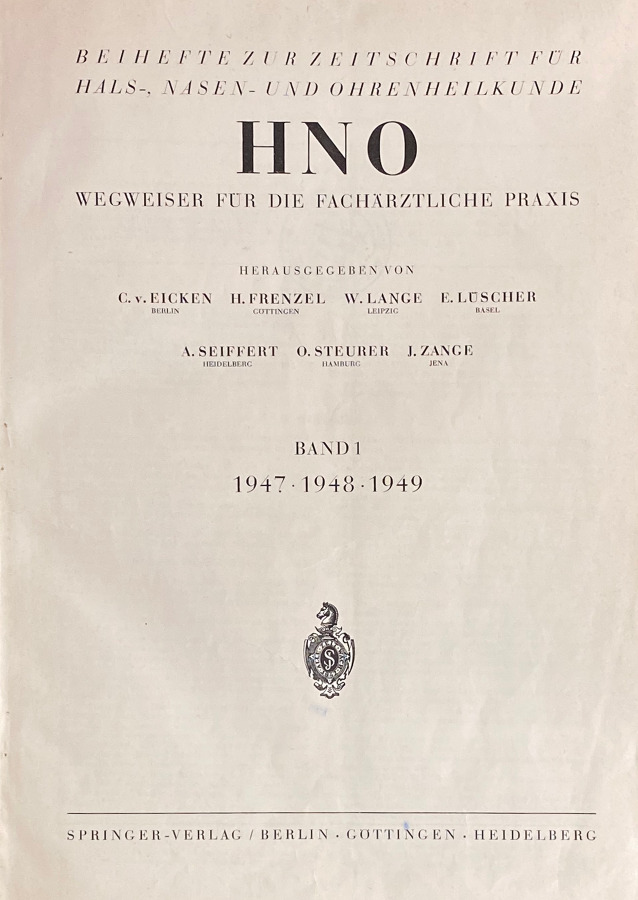

Die Titelhistorie der *HNO* ist recht geradlinig. Als Neugründung im Springer-Verlag nach dem 2. Weltkrieg sollte der Titel mit einem Fokus auf praktische Themen erscheinen. Die Zeitschrift *HNO/Organ der Deutschen Gesellschaft für Hals‑, Nasen- Ohrenheilkunde, Kopf- und Hals-Chirurgie* wurde vom Springer-Verlag 1947 zunächst als Beiheft zur *Zeitschrift für Hals-Nasen- und Ohrenheilkunde* gegründet. Obwohl diese Zeitschrift zuvor schon in das *Archiv für Ohren‑, Nasen- und Kehlkopfheilkunde *aufgegangen war, findet sich auf dem Cover noch dieser Zusatz. Am ehesten ist dies wohl auf den Neuanfang des Verlagswesens nach dem 2. Weltkrieg mit den anfangs schwierigen Lizenzvergaben in den westlichen Besatzungszonen zurückzuführen. In einem Brief von 1947 beklagt sich Ferdinand Springer über die Verweigerung der Lizenz für die wichtigsten und bekanntesten Zeitschriften seines Verlags, u. a. für das *Archiv für Ohren‑, Nasen- und Kehlkopfheilkunde (vereinigt mit Zeitschrift für Hals-Nasen-Ohrenheilkunde)*, durch die vorgesetzten amerikanischen Behörden (vgl. Götze, 1994, S. 4 [33]). Im Jahr 1958 fiel dann der Namenszusatz weg, und der Titel *HNO *erscheint seitdem als Publikationsorgan der Deutschen Gesellschaft für Hals-Nasen-Ohren-Heilkunde, Kopf- und Hals-Chirurgie und später auch der Deutschen Akademie für Hals-Nasen-Ohren-Heilkunde, Kopf- und Hals-Chirurgie unter gleichem Namen. Die Zeitschrift *HNO* trug in den ersten Jahren auch den Zusatz zum Sachtitel „Wegweiser für die fachärztliche Praxis“, was auf die eher klinisch-praktische Zielsetzung der Verleger und des ersten Herausgebers Hermann Frenzel (1895–1967) schließen lässt. Frenzel, 1925 bei Wilhelm Brünings in Greifswald für das Fach Hals-Nasen-Ohren-Heilkunde habilitiert, wurde 1942 als ordentlicher öffentlicher (o. ö.) Professor nach Göttingen berufen. In seinem Nachruf für Hermann Frenzel beschreibt Minnigerode 1968 die Beziehungen zwischen der *HNO* und dem *Archiv *in der Nachkriegszeit wie folgt: „Nach dem Rücktritt Seiferts, mit dem er sich gemeinsam um das Wiedererscheinen und die Neuorganisation unserer Fachzeitschrift durch Teilung in das der klinischen und experimentellen Forschung dienende Archiv und den auf die Fachpraxis ausgerichteten HNO-Wegweiser bemüht hatte, übernahm Hermann Frenzel 1955 die Schriftleitung des *Archivs für Hals-Nasen-Ohrenheilkunde*, dessen geschäftsführender Herausgeber er bis zu seinem Tode war“ [61]. Schon zu Beginn gilt die *HNO* als Publikationsorgan der Deutschen Gesellschaft für Hals-Nasen-Ohren-Heilkunde, Kopf- und Hals-Chirurgie, der Schweizerischen Gesellschaft für Oto-Rhino-Laryngologie, Hals- und Gesichtschirurgie (ab 1977) sowie mehrerer regionaler HNO-Fachgesellschaften, interessanterweise bis 1975 auch für die ostdeutschen medizinischen Gesellschaften für HNO-Heilkunde in Halle, Jena, Leipzig, Rostock und Greifswald. Im Jahr 2008 wird die *HNO* neues Mitteilungsorgan auch der Österreichischen HNO-Gesellschaft. Gemeinsame wissenschaftliche und berufspolitische Interessen beider Fachgesellschaften sollen starke Akzente in der europäischen Entwicklung des HNO-Fachs setzen [10]. Seit 2016 können in der *HNO* Beiträge zusätzlich zur deutschen Sprache auch in einer englischen Version in einem 2‑mal im Jahr online erscheinenden Supplementband veröffentlicht werden. Auch eine ausschließlich englischsprachige Publikation ist im Ausnahmefall möglich. In Tab. 2 sind die Herausgeber bzw. die Schriftführer der *HNO* seit 1947 aufgeführt. Die Abb. 6 zeigt das Titelblatt des ersten Bands der *HNO* von 1947/1948/1949.

**Table 2:** 

***HNO/Deutsche Gesellschaft für Hals-Nasen-Ohren-Heilkunde, Kopf- und Hals-Chirurgie, Deutsche Akademie für Hals-Nasen-Ohren-Heilkunde, Kopf- und Hals-Chirurgie***
Heidelberg: Springer-Verlag GmbH, Springer Medizin; Heidelberg: Springer-Medizin-Verl. **1.** 1947/49, Okt.–
**1.** 1947/49 – **7.** 1958/59 Beilage zu: Archiv für Ohren‑, Nasen- und Kehlkopfheilkunde (2 – Tab. 1)
Hrsg. (1947–): Carl von Eicken, Berlin, Hermann Frenzel, Göttingen, Wilhelm Lange, Leipzig, Erhard Lüscher, Basel, Alfred Seiffert, Heidelberg, Otto Steurer, Hamburg, Johannes Zange, Jena (Gründungsherausgeber), Kaarlo Y. A. Meurman, Helsinki, Max Schwarz, Tübingen, Siegfried Unterberger, Klagenfurt, Julius Berendes, Marburg, Rudolf Link, Hamburg, Adolf Miehlke, Göttingen, Rudolf Pfaltz, Basel, Franz Escher, Bern, Ernst Lehnhardt, Hannover, Hans-Peter Zenner, Tübingen, Peter-Karl Plinkert, Heidelberg, Stefan K. Plontke, Halle/Saale

### Titelhistorie *Laryngo-Rhino-Otologie: LRO*

#### *Organ der Deutschen Gesellschaft für Hals-Nasen-Ohren-Heilkunde, Kopf- und Hals-Chirurgie; Organ der Deutschen Akademie für Hals-Nasen-Ohren-Heilkunde, Kopf- und Hals-Chirurgie; Organ der Österreichischen Gesellschaft für Hals-Nasen-Ohren-Heilkunde, Kopf- und Hals-Chirurgie* Stuttgart; New York, NY: Thieme 68.1989 – [18]

**Figure 7:**
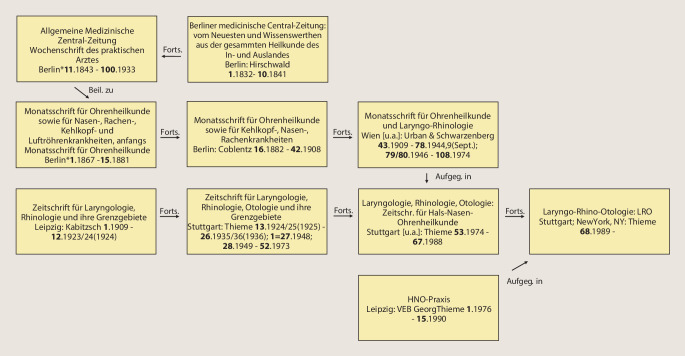

**Figure 8:**
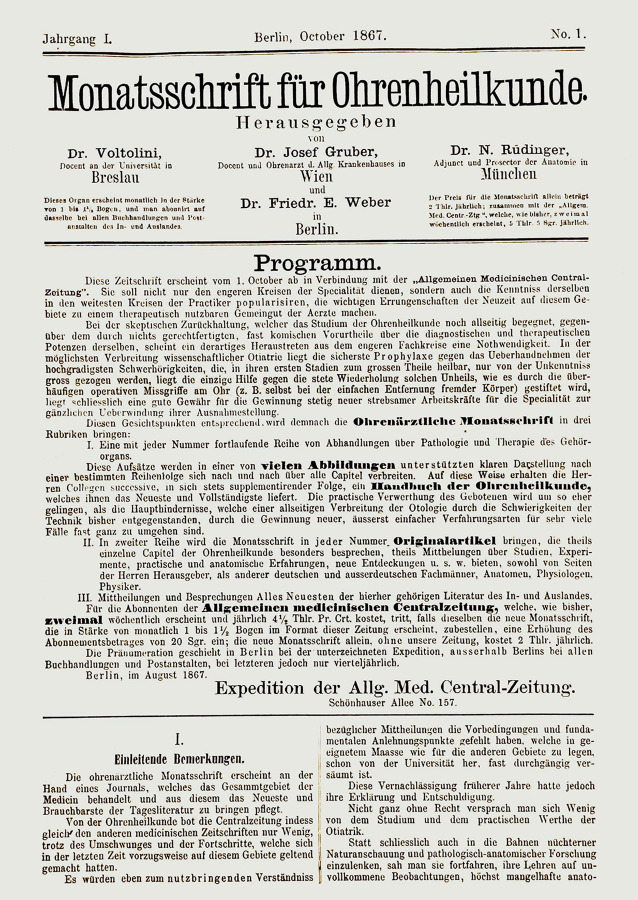

Die heute im Thieme-Verlag Stuttgart, New York, NY, erscheinende Zeitschrift *Laryngo-Rhino-Otologie: LRO* trug 2020 die Volume-Nummer 99. Sie geht auf 2 Zeitschriftengründungen um die vorletzte Jahrhundertwende zurück: die *Monatsschrift für Ohrenheilkunde* und die *Zeitschrift für Laryngologie, Rhinologie und ihre Grenzgebiete*. Folgt man den Volume-Nummern, wurde der erste Band 1909 in Leipzig bei C. Kabitzsch als *Zeitschrift für Laryngologie, Rhinologie und ihre Grenzgebiete* gegründet. Diese Zeitschrift wurde als *Zeitschrift für Laryngologie, Rhinologie, Otologie und ihre Grenzgebiete: Organ d. Vereinigung Südwestdeutscher Hals‑, Nasen‑, Ohrenärzte* nach dem 2. Weltkrieg bei Thieme in Stuttgart bis zum Volume 52 im Jahr 1973 fortgeführt. Die zweite Gründung geht auf das Jahr 1867 zurück. Die *Monatsschrift für Ohrenheilkunde sowie für Nasen‑, Rachen‑, Kehlkopf- und Luftröhrenkrankheiten, *anfangs* Monatsschrift für Ohrenheilkunde,* wurde bis 1881 als „Expedition der Allgemeinen Medizinischen Centralzeitung“ in Berlin und ab 1882 als *Monatsschrift für Ohrenheilkunde sowie für Kehlkopf‑, Nasen‑, Rachenkrankheiten: Organ d. Österreichischen Otologischen Gesellschaft u. d. Münchener Laryngo-Otologischen Gesellschaft* im Verlag Oscar Coblentz, Berlin, fortgeführt. Ab Volume 43 im Jahr 1909 erfolgte die Herausgabe durch den Verlag Urban Schwarzenberg in Wien als *Monatsschrift für Ohrenheilkunde und Laryngo-Rhinologie: Organ der Österreichischen Oto-Laryngologischen Gesellschaft und der Wiener Gesellschaft der Hals‑, Nasen‑, Ohren-Ärzte*. Letztgenannte Zeitschrift behielt ihre Selbstständigkeit bis zum Volume 108 und ging im Jahr 1974 in der Zeitschrift *Laryngologie, Rhinologie, Otologie* bei Thieme in Stuttgart auf. In der Abb. 7 ist der Verlauf der Titeländerungen und Fusionierungen in einem Flussdiagramm dargestellt. Die jeweiligen Titelblätter der neu gegründeten Vorgänger-Zeitschriften der *LRO* sind in den Abb. 8 und 9 dargestellt.

**Figure 9:**
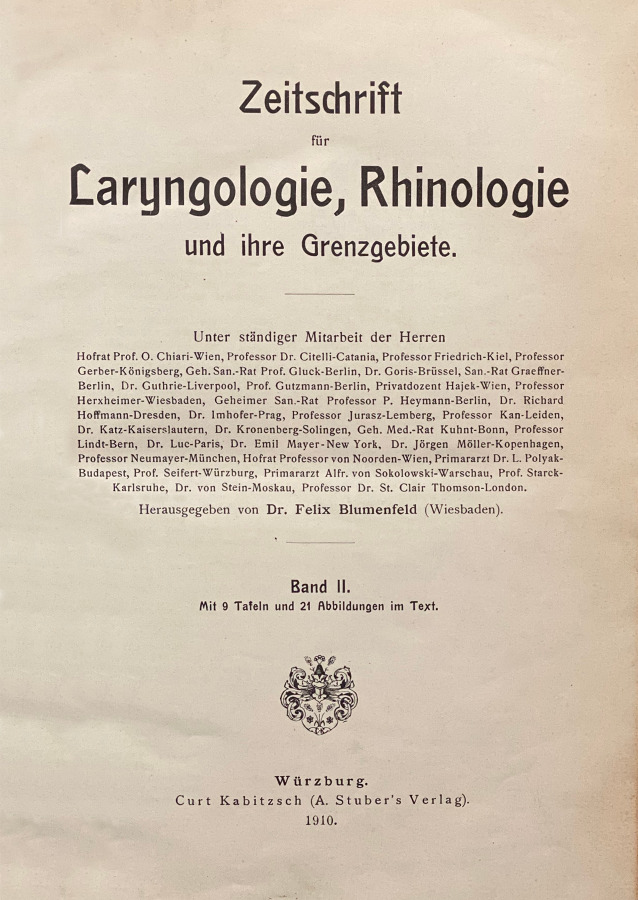

Zunächst wurde die *Monatsschrift für Ohrenheilkunde* als Expedition der *Allgemeinen Medizinischen Central-Zeitung* 1867 in Berlin gegründet. Diese Universalzeitschrift erschien von 1832 bis 1933 zunächst im Berliner Verlag Hirschwald als *Berliner medicinische Central-Zeitung*, dann im Selbstverlag des Gründers der Zeitschrift, Johann Jakob Sachs (1803–1846), und später im Berliner Verlag Oskar Coblentz [70]. Zum Zeitpunkt der Gründung der Monatsschrift gehörte der Verlag der Allgemeinen Central-Zeitung der Witwe des ersten Herausgebers, Fanny Sachs. Erste Herausgeber der Monatsschrift waren Rudolf Voltolini (1819–1889), Breslau, Josef Gruber (1827–1900), Wien, Nikolaus Rüdinger (1832–1896), München, und Friedrich Eugen Weber, Berlin, ab 1873 Weber-Liel (1832–1891). Weber hatte aus persönlichen Gründen den Geburtsnamen seiner Mutter seinem Namen hinzugefügt. Rüdinger war als Anatom in München tätig. Die 3 Otologen hatten sich um die Gründungszeit des Journals für die Otologie habilitiert, Voltolini 1862, Gruber 1863 und Weber 1872. Die Herausgeber beschreiben im Editorial des ersten Bands im Programm für die neue Zeitschrift 3 Ziele. Gegründet als Beilage zu einer klassischen Universalzeitschrift sollte das Blatt den praktizierenden Ärzten durch bebilderte Aufsätze einen Einblick in das Spezialfach der Otologie ermöglichen. Außerdem sollten Originalartikel den wissenschaftlichen Charakter unterstreichen und ferner Besprechungen der neuesten Literatur des In- und Auslandes erscheinen (vgl. Weber 1867 [101]). Diese 3 Funktionen des Titels haben sich in der weiteren historischen Entwicklung der wissenschaftlichen Zeitschriften oft in speziellen Journalen mit Schwerpunkt in wissenschaftliche Originalarbeiten, Fort- und Weiterbildung und insbesondere in den Referate-Zeitschriften (Zentralblätter) gefunden. Mittlerweile haben aber aktuelle Zeitschriften wie z. B. die *HNO* und *LRO* als Rubriken „Originalarbeiten“, „Vorbereitung auf die Facharztprüfung“ (Weiterbildung), „CME“ (Fortbildung) und „Update“. Eine inhaltliche Erweiterung der bis dahin überwiegend otologisch geprägten Zeitschrift erfolgte 1876 mit der Ergänzung des Zeitschriftentitels um „Nasen‑, Rachen‑, Kehlkopf- und Luftröhrenkrankheiten“, um den zunehmend ins Blickfeld geratenden Nachbarschaftsbeziehungen zum Rachen, zum Kehlkopf und zu den Atemwegen wissenschaftlich und klinisch gerecht zu werden. In Frankreich wurde jedoch schon ein Jahr zuvor, 1875, mit den *Annales des maladies de l’oreille et du larynx (otoscopie, laryngoscopie, rhinoscopie) *die erste die Gesamtheit des HNO-Fachgebiets umfassende Zeitschrift gegründet.

Bemerkenswert in der Historie der Monatsschrift war 1934 die Einführung eines Generalregisters. In der heutigen Zeit der Digitalisierung kann man kaum ahnen, welcher Aufwand mit der nachträglichen manuellen Schaffung eines solchen Registers betrieben werden musste. Da die Zeitschrift anfangs in anderen Verlagen erschien, entschloss sich der Verlag Urban Schwarzenberg, das Zentralregister ab Band 43 des Jahres 1909 bis zum Band 67 des Jahres 1933 zu erfassen. Es wurden nicht nur Autorennamen, sondern auch ein Sachregister eingeführt (vgl. Urbantschitsch 1934 [96]). Auch andere Zeitschriften, wie die *AfO*, führten in dieser Zeit aufwendige Generalregister [37]. Zum 100. Geburtstag der Monatsschrift beschrieb Fremel 1966 die historische Aufgabe der Zeitschrift als Bindeglied für den Vielvölkerstaat Österreich-Ungarn. Diese spezifisch österreichische Rolle konnte die Monatsschrift während der reichsdeutschen Gleichschaltung ab 1938 nicht mehr erfüllen (vgl. Fremel 1966 [31]). Erst mit der Gründung der Österreichischen Oto-Laryngologischen Gesellschaft 1945/46 unter seinem Präsidenten E. Urbantschitsch konnte die Zeitschrift wieder in ihrer alten Form erscheinen (vgl. [31]). Die Monatsschrift wurde unter dem Titel *Monatsschrift für Ohrenheilkunde und Laryngo-Rhinologie* bis zum Band 108, 1974, beim Verlag Urban Schwarzenberg weitergeführt und ist ab 1974 ab Band 53 in der Zeitschrift *Laryngologie, Rhinologie, Otologie* des Stuttgarter Thieme-Verlags aufgegangen. Allerdings erscheint in der LRO erst 1976 ab Band Nr. 55 der offizielle Zusatz „… vereinigt mit Monatsschrift für Ohrenheilkunde“. Über die Gründe des Aufgehens der Monatsschrift in der LRO finden sich weder Hinweise in den Editorials beider Zeitschriften zu dieser Zeit, noch wird hierüber in den Jubiläumsschriften der Verlage berichtet. Es ist anzunehmen, dass dem Übergang der Monatsschrift vom Verlag Urban Schwarzenberg zum Thieme-Verlag betriebswirtschaftliche Gründe in den Verlagen und Kostengründe der Fachgesellschaft zugrunde gelegen haben. Von 1909 bis 1974 führten nacheinander insgesamt 17 Herausgeber die Zeitschrift (Tab. 3).

**Table 3:** 

1	***Monatsschrift für Ohrenheilkunde sowie für Nasen‑, Rachen‑, Kehlkopf- und Luftröhrenkrankheiten, anfangs Monatsschrift für Ohrenheilkunde***
	Berlin: Expedition der Allgemeinen Medizinischen Zentralzeitung **1**. 1867 – **15**. 1881
	Hrsg. (1867–1881): Rudolf Voltolini, Breslau, Josef Gruber, Wien, Nikolaus Rüdinger, München, Friedrich Eugen Weber, Berlin (Gründungsherausgeber), Leopold von Schrötter, Wien, Max-Joseph Oertel, München
2	***Monatsschrift für Ohrenheilkunde sowie für Kehlkopf‑, Nasen‑, Rachenkrankheiten: Organ der Österreichischen Otologischen Gesellschaft u. d. Münchener Laryngo-Otologischen Gesellschaft***
	Berlin: Coblentz **16**. 1882 – **42**. 1908
	Hrsg. (1882–1908): Rudolf Voltolini, Breslau, Josef Gruber, Wien, Nikolaus Rüdinger, München, Friedrich Eugen Weber, Berlin, Leopold von Schrötter, Wien, Max-Joseph Oertel, München, Michael Joseph Rossbach, Würzburg, Philip Schech, München, Emil Zuckerkandl, Wien, Viktor Urbantschitsch, Wien, Antoni Jurasz, Heidelberg
3	***Monatsschrift für Ohrenheilkunde und Laryngo-Rhinologie: Organ der Österreichischen Oto-Laryngologischen Gesellschaft und der Wiener Gesellschaft der Hals‑, Nasen‑, Ohren-Ärzte***
	Wien [u. a.]: Urban Schwarzenberg **43**. 1909 – **78**. 1944, 9 (Sept.); **79/80**. 1946 – **108**. 1974, Aufgeg. in: Laryngologie, Rhinologie, Otologie
	Hrsg. (1909–1944): Emil Zuckerkandl, Wien, Viktor Urbantschitsch, Wien, Antoni Jurasz, Lemberg, Ottokar Chiari, Wien, Heinrich Neumann, Wien, Markus Hajek, Wien, Gustav Alexander, Wien, Gustav Hofer, Graz, Wilfried Krainz, Innsbruck, Hermann Marschik, Wien, Ernst Urbantschitsch, Wien, Siegfried Unterberger, Wien, Emil A. Wessely, Wien
	Hrsg. (1946–1974): Ernst Urbantschitsch, Wien, Otto Novotny, Wien, Friedrich Krejci, Wien, Eduard H. Majer, Wien, Kurt Burian, Wien
4	***Zeitschrift für Laryngologie, Rhinologie und ihre Grenzgebiete***
	Leipzig: Kabitzsch **1**. 1909 – **12**. 1923/24(1924), Forts.: Zeitschrift für Laryngologie, Rhinologie, Otologie und ihre Grenzgebiete
	Hrsg. (1909–1924): Felix Blumenfeld, Wiesbaden
5	***Zeitschrift für Laryngologie, Rhinologie, Otologie und ihre Grenzgebiete: Organ d. Vereinigung Südwestdeutscher Hals‑, Nasen‑, Ohrenärzte***
	Stuttgart: Thieme **13.** 1924/25 (1925) – **26.** 1935/36 (1936); **1** **=** **27**. 1948; **28.** 1949 – **52.** 1973, Forts.: Laryngologie, Rhinologie, Otologie
	Hrsg. (1924–1936): Felix Blumenfeld, Wiesbaden, Alexander Herrmann, Erfurt
	Hrsg. (1948–1973): Hans Leicher, Stuttgart/Mainz, Max Meyer, Würzburg, Richard Mittermaier, Marburg/Frankfurt/M, Gerhard Theissing, Ludwigshafen/Erlangen, Walter Becker, Bonn, Hans-Georg Boenninghaus, Heidelberg
6	***Laryngologie, Rhinologie, Otologie: Zeitschr. für Hals-Nasen-Ohrenheilkunde; Organ der Deutschen Gesellschaft für Hals-Nasen-Ohren-Heilkunde, Kopf- und Halschirurgie; Organ der Österreichischen Gesellschaft für Hals-Nasen-Ohren-Heilkunde, Kopf- und Halschirurgie***
	Stuttgart [u. a.]: Thieme **53**. 1974 – **67**. 1988, Forts.: Laryngo-Rhino-Otologie
	Hrsg. (1974–1988): Hans-Georg Boenninghaus, Heidelberg, Hans-Heinz Naumann, München, Kurt Burian, Wien, Harald Feldmann, Münster
7	***Laryngo-Rhino-Otologie: LRO: Organ der Deutschen Gesellschaft für Hals-Nasen-Ohren-Heilkunde, Kopf- und Hals-Chirurgie; Organ der Deutschen Akademie für Hals-Nasen-Ohren-Heilkunde, Kopf- und Hals-Chirurgie; Organ der Österreichischen Gesellschaft für Hals-Nasen-Ohren-Heilkunde, Kopf- und Hals-Chirurgie***
	Stuttgart; New York, NY: Thieme **68**. 1989–
	Hrsg. (1989–): Harald Feldmann, Münster, Kurt Burian, Wien, Ernst Kastenbauer, München, Heinz Stammberger, Graz, Heinrich Rudert, Kiel, Jan Helms, Würzburg, Gerhard Rettinger, Ulm, Orlando Guntinas-Lichius, Jena, Andreas Dietz, Leipzig, Stefan Dazert, Bochum, Gerd Rasp, Salzburg

Die *Zeitschrift für Laryngologie, Rhinologie und ihre Grenzgebiete* wurde 1909 im Verlag Curt Kabitzsch in Würzburg gegründet. Betrachtet man die Zählung der Bände, so wird sie bis heute (2020) mit Volume 99 als LRO beim Thieme-Verlag, Stuttgart, fortgeführt. Gründungsherausgeber war Felix Blumenfeld (1864–1947) aus Wiesbaden. Im Editorial des ersten Bands nimmt der Herausgeber Bezug auf die Einführung des Kehlkopfspiegels durch Türck und Czermak 50 Jahre zuvor und deren Würdigung auf dem ersten internationalen Laryngologen-Kongress 1908 in Wien. Die Einführung der Laryngoskopie in die Klinik wird als große Errungenschaft und als Beginn einer wissenschaftlichen Laryngologie gewürdigt (vgl. Blumenfeld 1909 [9]). Die neue Zeitschrift solle dem weiteren Ausbau dieses Fachgebiets und den Bedürfnissen des praktischen Hals-Nasen-Ohren-Arztes dienen. Blumenfeld betrieb über Jahrzehnte eine große fachärztliche Praxis im Kurort Wiesbaden und war auch wegen seiner umfangreichen publizistischen Tätigkeit weit über die Grenzen seiner Heimatregion bekannt. Er war Mitherausgeber mehrerer Standardwerke zur Chirurgie, Pathologie und auch zur Tuberkulose. Bis zum durch die Nationalsozialisten erzwungenen Entzug seiner Herausgeberschaft 1934 war er ununterbrochen Herausgeber der *Zeitschrift für Laryngologie, Rhinologie und ihre Grenzgebiete* (vgl. Schriftleitung und Verlag 1948 [80]). Einzige Änderung war die Ergänzung des Titels der Zeitschrift um den Begriff „Otologie“ im Jahr 1925, was mit der mittlerweile vollzogenen Vereinigung des Fachgebiets zu erklären sein dürfte. Das hohe Ansehen von Blumenfeld und der Zeitschrift kommen in der auf dem jeweiligen Cover erwähnten Mitarbeit zahlreicher Fachvertreter aus dem In- und Ausland zum Ausdruck. Obwohl keine eindeutigen Quellenangaben zum Entzug der Herausgeberschaft von Felix Blumenfeld und zu Stellungnahmen hierzu gefunden werden konnten, ist anzunehmen, dass das internationale Ansehen der deutschen HNO und das des Verlags hierunter deutlich gelitten haben dürften. Durch den Curt Kabitzsch-Verlag, der 1917 vom Leipziger Verlag J. A. Barth übernommen wurde, wurde dann auch der Name der Zeitschrift soweit geändert, dass der letzte Vorkriegsband 1936 mit der Nummer 26 als Teil 1 der *Folia oto-laryngologica* unter der Herausgeberschaft von Alexander Herrmann (1900–1981), Erfurt, erschien. Erst nach dem Krieg konnte die *Zeitschrift für Laryngologie, Rhinologie Otologie und ihre Grenzgebiete *im Thieme-Verlag als Neugründung zunächst als Band 1 erscheinen. Später erhielt „… dieser Jahrgang die Bezeichnung Band 27 im Anschluss an die frühere Zeitschrift für Laryngologie, Rhinologie Otologie“ [98]. Dies dürfte 1948 noch mit Vorgängen bei den Lizenzerteilungen durch die Besatzungsbehörden erklärt werden können. Erste Herausgeber nach dem Krieg waren Hans Leicher (1898–1989), Stuttgart, Max Meyer (1890–1954), Würzburg, Richard Mittermaier (1897–1983), Marburg, Gerhard Theissing (1903–1987), Ludwigshafen/Erlangen, und Walter Becker (1920–1990), Bonn. Ab Band 53 im Jahr 1971 wurde neben den Herausgebern und einem Beirat auch der Titel eines Schriftleiters auf dem Cover der Zeitschrift aufgeführt. Langjährige Schriftleiter waren Hans-Georg Boenninghaus (1921–2005), Heidelberg, Hans-Heinz Naumann (1919–2001), München, Kurt Burian (1921–1996), Wien, Harald Feldmann (1926–), Münster, Ernst Kastenbauer (1937–2004), München, Heinz Stammberger (1946–2018), Graz, Heinrich Rudert (1935–), Kiel, Jan Helms (1937–), Würzburg, Gerhard Rettinger (1947–), Ulm, und Orlando Guntinas-Lichius (1967–), Jena. Der aktuelle federführende Schriftleiter der LRO, Andreas Dietz (1962–), Leipzig wird von 3 weiteren Schriftleitern (Stefan Dazert (1963–), Bochum, Orlando Guntinas-Lichius, Jena, Gerd Rasp (1960–), Salzburg), 11 Rubrikenherausgebern, 29 Herausgebern, 11 Editores emeriti und 8 Beiratsmitgliedern beraten.

### Titelhistorie *HNO-Praxis*

#### *Hrsg.: Gesellschaft für Oto-rhino-laryngologie und Zerviko-faziale Chirurgie der DDR *Leipzig: VEB Georg Thieme [20]

**Figure 10:**
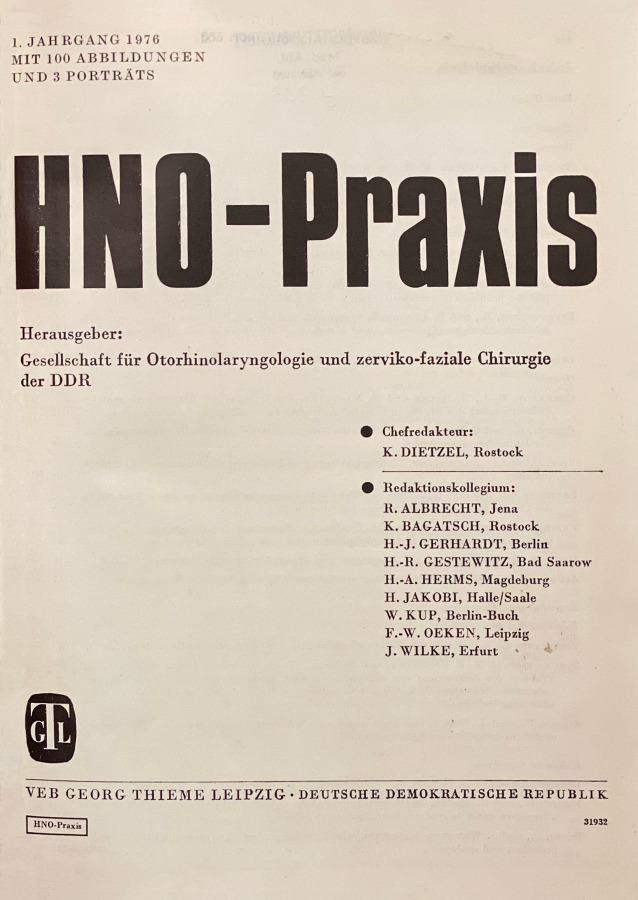

Die Historie der *HNO-Praxis* ist recht geradlinig (Tab. 4). Sie wurde 1976 beim Verlag VEB Georg Thieme Leipzig gegründet (Abb. 10) und 1980 um den Titel *Folia broncho-oesophagologica* ergänzt. Die Zeitschrift ging nach der Wiedervereinigung Deutschlands nach 1990 in der LRO auf.

**Table 4:** 

***HNO-Praxis/Hrsg.: Gesellschaft für Oto-rhino-laryngologie und Zerviko-faziale Chirurgie der DDR***
Leipzig: VEB Georg Thieme **1.** 1976 – **15.** 1990
Hrsg. (1976–1990): Kurt Dietzel, Rostock, Hans-Jürgen Gerhard, Berlin, Lutz Kessler, Dresden

Wegen der steigenden Zahl von HNO-Fachärzten in der Deutschen Demokratischen Republik (DDR) und dem gewachsenen wissenschaftlichen Potenzial an den Hochschulkliniken erfolgte 1976 im Auftrag des Generalsekretariats der medizinisch-wissenschaftlichen Gesellschaften beim Ministerium für Gesundheitswesen der DDR auf Antrag der Gesellschaft für Oto-rhino-laryngologie und Zerviko-faziale Chirurgie der DDR die Gründung einer wissenschaftlichen HNO-Fachzeitschrift. Scholz beschrieb 2000 die spezielle Situation der Medizin in der DDR zu dieser Zeit wie folgt: „Die Maßnahmen für eine Stärkung der DDR mit ständig steigender Abgrenzung gegenüber der Bundesrepublik sollten mit einer parallel angestrebten internationalen Anerkennung einhergehen. Die wissenschaftspolitischen Folgen für die Medizin waren der von der SED [Sozialistische Einheitspartei Deutschlands] geforderte Austritt der DDR-Ärzte aus westdeutschen Fachgesellschaften, die Gründung von Fachgesellschaften in der DDR mit der Durchführung eigener Kongresse, die Herausgabe von Zeitschriften als Publikationsorgane vorwiegend für DDR-Wissenschaftler“ [79]. Erster Herausgeber der *HNO-Praxis* war Kurt Dietzel (1912–2002), Rostock, ihm folgten Hans-Jürgen Gerhard (1928–2010), Berlin, und Lutz Keßler (1936–), Dresden. Kurt Dietzel, 1954 in Leipzig bei Woldemar Tonndorf (1887–1957) habilitiert, war zwischen 1961 und 1978 ordentlicher Professor für Oto-Rhino-Laryngologie in Rostock. Es ist das Verdienst Dietzels, die Zeitschrift 1980 um die Folia broncho-oesophagologica erweitert zu haben, signalisiert dies doch das Interesse der HNO-Heilkunde an den wichtigen endoskopischen Grenzgebieten, in Zeiten, als die Endoskopien des Ösophagus und des Tracheobronchialsystems überwiegend noch mit starren Rohrendoskopen durchgeführt wurden. In der letzten Ausgabe erfolgte ein nüchterner „Hinweis an unsere Abonnenten der Zeitschrift HNO-Praxis!“, dass die Zeitschrift zum Januar 1991 mit der Zeitschrift *Laryngo-Rhino-Otologie* des Georg Thieme Verlags, Stuttgart, vereint wird [38]. Allerdings wird in der LRO erst ab 1994 der bei Zeitschriftenvereinigungen übliche Zusatz „… vereint mit HNO-Praxis …“ erwähnt, was mit der Übernahme des VEB Georg Thieme Verlags (Ost), Leipzig, durch den Georg Thieme-Verlag (West), Stuttgart, und damit der Übernahme der Zeitschrift *HNO-Praxis* der Gesellschaft für Oto-rhino-laryngologie und Zerviko-faziale Chirurgie der DDR zusammenhängen dürfte. Die DDR-HNO-Ärzte wurden jedoch sofort als Abonnenten wahrgenommen. In der Zeit der Wiedervereinigung beider deutscher Staaten herrschte eine große Unsicherheit von Seiten der „HNO-Gemeinde“ der Bundesrepublik über eine mögliche Systemnähe der DDR-Ordinarien. Erst 1993 wurde mit Bernd Freigang (1941–), Berlin, wieder ein DDR-Professor in das Herausgebergremium der LRO gewählt.

### Titelhistorie *ORL: journal for oto-rhino-laryngology and its related specialities*

#### Einzelne Bd. Verhandlungsberichte der wissenschaftlichen Frühjahrsversammlung der Schweizerischen Gesellschaft für Otorhinolaryngologie, Hals- und Gesichtschirurgie, Einzelne Bd. von Nederlandse Keel-Neus-Oorheelkundige Vereniging: Vergadering, Basel; Freiburg, Br.; Paris; London; New York, NY; New Delhi; Bangkok; Singapore; Tokyo; Sydney: Karger [19]

In Berlin wurde 1908 im Samuel Karger-Verlag die Zeitschrift *Passow-Schäfer Beiträge zur Anatomie, Physiologie, Pathologie und Therapie des Ohres, der Nase und des Halses* gegründet (Tab. 5). Der Titel wurde bis zum Band 31 im Jahr 1934 herausgegeben. Die Gründungsherausgeber, der Otologe Adolf Passow (1859–1926), Berlin, und der Physiologe Karl Ludolf Schäfer (1866–1931), Berlin, konnten durch einen streng wissenschaftlichen Inhalt Autoren der HNO aus der ganzen Welt gewinnen. Beide arbeiteten eng zusammen, seit Schäfer 1907 Leiter des Akustisch-Physiologischen Laboratoriums der Ohrenklinik in Berlin wurde.

**Table 5:** 

1	***Beiträge zur Anatomie, Physiologie, Pathologie und Therapie des Ohres, der Nase und des Halses***
	Berlin: Karger **1**. 1908 – **24**. 1926
	Hrsg. (1908–1926): Adolf Passow, Berlin, Karl Ludolf Schaefer, Berlin
2	***Passow-Schäfer Beiträge zur Anatomie, Physiologie, Pathologie und Therapie des Ohres, der Nase und des Halses***
	Berlin: Karger **25**. 1927 – **31**. 1934/35 [?]
	Hrsg. (1927–1935): Alfred Güttich, Greifswald, Karl Ludolf Schaefer, Berlin, Oskar Wagener, Göttingen, Johannes Zange, Jena
3	***Practica oto-rhino-laryngologica: internat. Zeitschrift für Hals‑, Nasen‑, Ohrenheilkunde***
	Basel [u. a.]: Karger **1**. 1938 – **33**. 1971,6
	Hrsg. (1938–1971): Joseph Berberich, Frankfurt/M/New-York, NY, Emil Schlittler, Basel, Georges Canuyt, Strasbourg, Georg Kelemen, Budapest, Robert Lund, Kopenhagen, Vincenco Tanturri, Mailand (Gründungsherausgeber), Luzius Rüedi, Zürich, Eelco Huizinga, Groningen, Carl Rudolf Pfaltz, Basel
4	***ORL: journal for oto-rhino-laryngology and its related specialities***
	Basel; Freiburg, Br.; Paris; London; New York; New Delhi; Bangkok; Singapore; Tokyo; Sydney: Karger **34**. 1972–
	Hrsg. (1972–): Carl Rudolf Pfaltz, Basel, Wolfgang Arnold, Luzern/München, Bert W. O’Malley, Jr. Philadelphia, PA, Daqing Li, Philadelphia, PA

**Figure 11:**
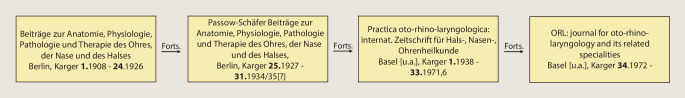

**Figure 12:**
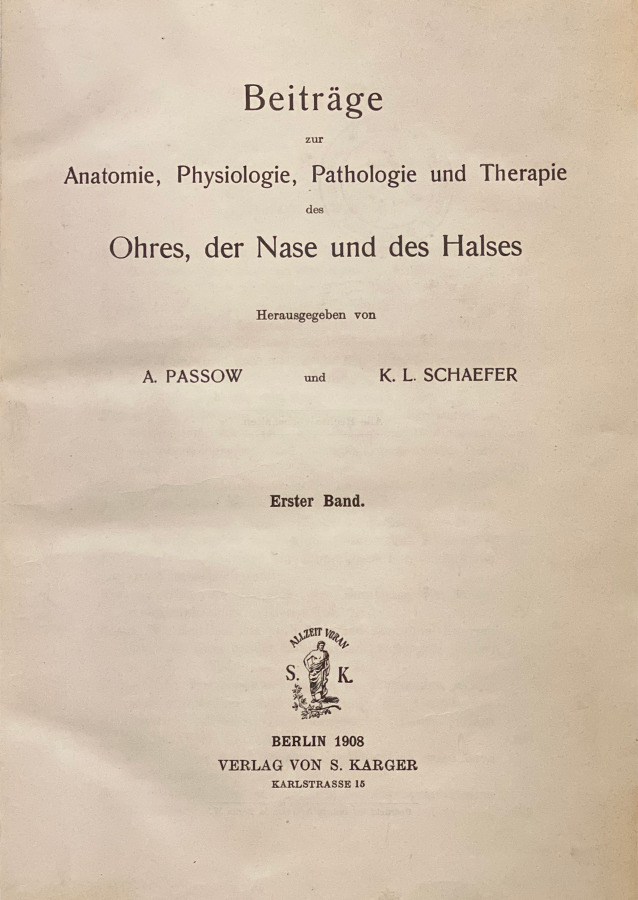

Mit dem durch die Nationalsozialisten erzwungenen Umzug des Verlags S. Karger nach Basel wurde die Zeitschrift zunächst eingestellt. Mit direktem Bezug auf die *Passow-Schäfer Beiträge* folgte als Fortsetzung und Neugründung mit neuer Zählung der Bände die dann mehr international und mehrsprachig ausgerichtete Zeitschrift *Practica oto-rhino-laryngologica: internat. Zeitschrift für Hals‑, Nasen‑, Ohrenheilkunde*, welche 1972 in* ORL: journal for oto-rhino-laryngology and its related specialities* umbenannt wurde. Der Titel wird bis heute mehrsprachig vom Verlag S. Karger, Basel, publiziert. Der Verlauf der Titeländerungen und Neugründungen ist in der Abb. 11 in einem Flussdiagramm dargestellt. Die jeweiligen Titelblätter der neu gegründeten Vorgängerzeitschriften der *ORL* sind in den Abb. 12 und 13 dargestellt.

**Figure 13:**
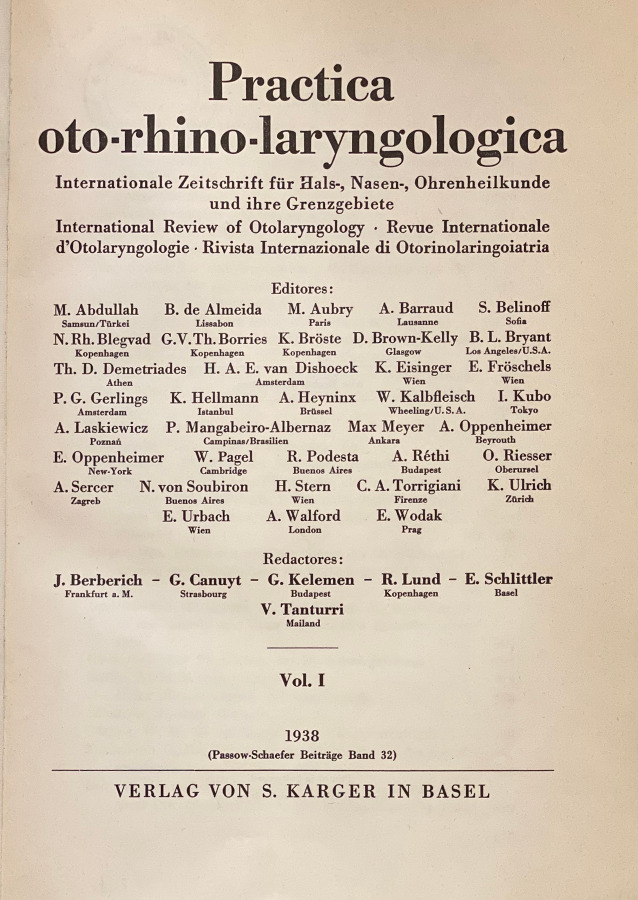

Durch die Verbote der Nationalsozialisten verlor der Verlag die Mehrzahl seiner Autoren und Herausgeber für die *Passow-Schäfer Beiträge* in Deutschland. Die Zeitschrift wurde mit dem Verlagsumzug nach Basel eingestellt. In der Einführung zum Eröffnungsheft (Vol. 1, 1938) der vom Karger Verlag, Basel, herausgegebenen Zeitschrift *Practica oto-rhino-laryngologica* bezieht sich der Verleger Samuel Karger explizit darauf, die neue HNO-Zeitschrift in der Tradition der 1908 im eigenen Verlag noch in Berlin gegründeten Zeitschrift *Passow-Schäfer Beiträge zur Anatomie, Physiologie, Pathologie und Therapie des Ohres, der Nase und des Halses* zu sehen. Sichtbar wurde dies durch den Zusatz „Passow-Schäfer Beiträge“ zum Zeitschriftentitel [47]. Auch die schon erwähnte Publikationsmöglichkeit für andere Sprachen wurde hier eingeführt [47]. Die Zeitschrift ist seit 1963 Publikationsorgan der Niederländischen HNO-Gesellschaft (Nederlandse Keel-Neus-Oorheelkundige Vereniging) und wurde im Jahr 1972 als Zeitschrift *ORL: journal for oto-rhino-laryngology and its related specialities* durch den S. Karger-Verlag, Basel, weitergeführt. Die ORL erscheint bis heute, 2020, im Volume 82, und publiziert regelmäßig auch die Verhandlungsberichte der wissenschaftlichen Frühjahrsversammlung der Schweizerischen Gesellschaft für Otorhinolaryngologie, Hals- und Gesichtschirurgie [19].

Um die vorletzte Jahrhundertwende entstanden auch zahlreiche Referateorgane, welche für den Kliniker und den praktisch tätigen Arzt die zunehmende Zahl von Publikationen weltweit zusammenfassten und referierten sowie Kongressberichte herausgaben. Neben dem Herausgeber waren hier zahlreiche Otologen, Laryngologen und Rhinologen als Übersetzer tätig, um Originalarbeiten aus anderen Sprachen ins Deutsche zu übertragen. Die Referateorgane hielten sich viele Jahrzehnte und wurden mit der zunehmenden Dominanz des Englischen als Wissenschaftssprache später eingestellt. Heute lebt diese Tradition als Rubrik in einigen aktuellen HNO-Publikationen fort, wird in Form von Literaturzirkeln in den Kliniken oder in privatwirtschaftlich organisierten Update-Veranstaltungen fortgeführt.

### Titelhistorie *Internationales Centralblatt für Laryngologie, Rhinologie und verwandte Wissenschaften*

#### Berlin: Hirschwald 1. 1884/85 – 25. 1909,6, [17]

Das älteste deutschsprachige HNO-Referateorgan war das 1884 gegründete *Internationale Centralblatt für Laryngologie, Rhinologie*. Bis 1922 erschien der Titel im Hirschwald-Verlag, Leipzig. Mit der Übernahme durch den Springer-Verlag erfolgte eine neue Zählung der Bände und die Ergänzung durch die Otologie. Bis zu seiner Einstellung mit Band 148 im Jahr 1996 erfolgten noch diverse Namensänderungen. Der erste Herausgeber war der deutsch-britische Laryngologe Felix Semon (1849–1921). Er hatte zahlreiche Laryngologen aus Europa und Nordamerika zur Mitarbeit gewonnen, auch um Veröffentlichungen in den meisten europäischen Sprachen in deutscher Übersetzung referieren zu können. Der Titelverlauf, die Umbenennungen und die Herausgeber sind in Tab. 6 dargestellt. Felix Semon, 1849 in Danzig geboren, hatte nach dem Medizinstudium in Heidelberg und Berlin seine Ausbildung beim damals bekanntesten englischen Laryngologen, Morell Mackenzie, in London begonnen und dann auch schnell als Sekretär der Sub-Sektion Laryngologie des Internationalen Medizin-Kongresses eine wissenschaftliche Karriere gemacht [94]. Er galt später als einer der bekanntesten Laryngologen weltweit, lebte und wirkte von nun an in London, wurde britischer Staatsbürger und 1897 von Queen Victoria in den Ritterstand erhoben. Er führte das *Internationale Centralblatt für Laryngologie, Rhinologie und verwandte Wissenschaften *ein Vierteljahrhundert lang. Der in deutscher Sprache erscheinende Titel war zu dieser Zeit das einzige internationale Journal für Rhinologie und Laryngologie. Als Referateblatt wurden jährlich bis zu 2000 Publikationen aus bis zu 15 Ländern Europas und Nordamerikas besprochen [28]. Wegen seiner großen Verdienste um die Zeitschrift entschieden der Verleger August Hirschwald und der neue Herausgeber Georg Finder 1909 nach dem Rückzug Semons als Herausgeber die Umbenennung in *Semon’s Internationales Centralblatt für Laryngologie, Rhinologie und verwandte Wissenschaften.* Unglücklicherweise kam es mit Beginn des 1. Weltkriegs und der damit verbundenen Verbreitung nationalistischer Tendenzen in der Gesellschaft im Jahr 1915 zu einem Zerwürfnis zwischen Verleger und Herausgeber auf der einen Seite und dem Begründer der Zeitschrift auf der anderen Seite. Nach einer öffentlichen Erklärung Semons in *The Times* zu den „… barbarous methods, one and all, employed by Germany“ und einer im *Centralblatt* veröffentlichen Erwiderung von Finder und Hirschwald mit der Entscheidung der Umbenennung der Zeitschrift ohne den Namenszusatz Semon’s kam es zu einer Polarisierung der Laryngologen-Community in Europa und Nordamerika. Eine Reihe von britischen und amerikanischen Mitherausgebern zog sich aus der Zeitschrift zurück, verschiedene deutsche, österreichische und ungarische Fachgesellschaften zogen ihre Ehrenmitgliedschaften für Semon zurück. Der Vorgang wird in einem Kommentar in *The Lancet *von 1915 beschrieben und die Namensänderung des so bedeutenden internationalen Journals als boshafte Kompetenzüberschreitung des Verlegers Hirschwald kritisiert (vgl. *The Lancet* 1915, S. 404–405 [4]). Das *Internationales Centralblatt für Laryngologie, Rhinologie und verwandte Wissenschaften *kam durch die Übernahme des Hirschwald-Verlags zu Springer im Jahr 1921 und wurde als Organ der wissenschaftlichen HNO-Fachgesellschaft auch nach dem 2. Weltkrieg weiter betrieben und diente überwiegend der Publikation der Inhalte seiner Jahresversammlungen. Nach diversen Umbenennungen wurde das Erscheinen dann 1996 eingestellt.

**Table 6:** 

1	***Internationales Centralblatt für Laryngologie, Rhinologie und verwandte Wissenschaften***
	Berlin: Hirschwald** 1**. 1884/85 – **25**. 1909, 6
	Hrsg. (1884–1909): Felix Semon, London
2	***Semon’s internationales Centralblatt für Laryngologie, Rhinologie und verwandte Wissenschaften***
	Berlin: Hirschwald **25**. 1909,7 –** 31**. 1915, 5
	Hrsg. (1909–1915): Georg Finder, Berlin
3	***Internationales Zentralblatt für Laryngologie, Rhinologie und verwandte Wissenschaften***
	Berlin: Hirschwald **31**. 1915,6 –**38**. 1922, 3
	Hrsg. (1915–1922): Georg Finder, Berlin
4	***Zentralblatt für Hals‑, Nasen- und Ohrenheilkunde sowie deren Grenzgebiete: Organ der Deutschen Gesellschaft der Hals‑, Nasen‑, Ohrenärzte***
	Berlin: Springer **1**. 1922 – **110**. 1975, 5 (Sept.)
	Hrsg. (1922–1975): Georg Finder, Berlin, Alfred Güttich, Berlin/Köln, Karl L. Schaefer, Berlin, Hermann Beyer, Berlin/Münster, Artur Blohmke, Frankfurt a. M., Carl v. Eicken, Berlin, Max Meyer, Würzburg, Hermann Frenzel, Göttingen, Werner Kindler, Berlin/Heidelberg, Alfred Seiffert, Heidelberg, Otto Steurer, Hamburg, Woldemar Tonndorf, Dresden/Leipzig, Rudolf Link, Berlin/Hamburg, Otto Novotny, Wien, Luzius Rüedi, Zürich, Hans-Jürgen Denecke, Heidelberg
5	***Zentralblatt für Hals‑, Nasen- und Ohrenheilkunde, plastische Chirurgie an Kopf und Hals: Organ der Deutschen Gesellschaft für Hals-Nasen-Ohrenheilkunde, Kopf- und Halschirurgie***
	**110**. 1975,6 (Dez.) (1976); **111**. 1975/76 (1975/77), 1 (Okt.) – **127**. 1981
	Hrsg. (1975–1981): Rudolf Link, Hamburg, Otto Novotny, Wien, Luzius Rüedi, Zürich, Hans-Jürgen Denecke, Heidelberg
6	***Zentralblatt Hals-Nasen-Ohrenheilkunde, plastische Chirurgie an Kopf und Hals: Organ d. Deutschen Gesellschaft für Hals-Nasen-Ohrenheilkunde, Kopf- und Halschirurgie*** ***=*** ***Oto-Rhino-Laryngology, plastic surgery of head and neck***
	Berlin; Heidelberg [u. a.]: Springer **128**. 1982 – **148**. 1996; damit Ersch. eingest.
	Hrsg. (1982–1996): Rudolf Link, Hamburg, Otto Novotny, Wien, Luzius Rüedi, Zürich, Hans-Jürgen Denecke, Heidelberg

### Titelhistorie *Internationales Centralblatt für Ohrenheilkunde*

#### Leipzig: J. A. Barth, 1. 1903 – 5. 1907, [35]

Im Jahr 1903 wurde vom Verlag J. A. Barth, Leipzig, ein weiteres Referateorgan gegründet, welches sich zunächst ausschließlich der Aufarbeitung der internationalen otologischen Literatur widmete. Gründungsherausgeber waren Oskar Brieger (1864–1914), Breslau, und Giuseppe Gradenigo (1859–1926), Turin. Brieger war zu dieser Zeit Primärarzt am Allerheiligenhospital in Breslau. Er veröffentlichte selbst zu otologischen Themen, wie Mittelohrtuberkulose, die operative Behandlung chronischer Mittelohrentzündungen und zu otogenen Erkrankungen der Hirnhäute. Gradenigo arbeitete bei Politzer in Wien und avancierte 1889 zum Direktor der Universitätsklinik für Ohren- und Kehlkopfkranke in Turin. Als Mitbegründer der *Archivio italiano di Otologia, Rhinologia e Laryngologia* [29] überblickte er die italienischsprachigen Veröffentlichungen im HNO-Fachgebiet. Weitere 15 Otologen aus Europa, aus Nord- und Südamerika konnten zur Mitarbeit am ersten Band des *Centralblatts* gewonnen werden, sodass damit auch Veröffentlichungen in allen zu dieser Zeit wichtigen Sprachen, wie Englisch, Französisch, Spanisch, Russisch und den skandinavischen Sprachen in deutscher Übersetzung referiert werden konnten. Schon 1913 wurde die Rhinologie und Laryngologie mit einbezogen. Über den langen Zeitraum von 22 Jahren, von 1913 bis 1935, war Franz Kobrak (1879–1955), Berlin, Mitherausgeber des *Internationalen Zentralblatts für Ohrenheilkunde und Rhino-Laryngologie*. Der aus Breslau stammende Kobrak hatte nach seinem Studium in Breslau und München seine Fachausbildung bei Brieger in Breslau absolviert und sich 1907 als Facharzt in Berlin niedergelassen. Nach seiner Habilitation an der Ohrenklinik der Charité bei Passow erfolgte 1926 die Ernennung zum außerordentlichen Professor an der Charité. Er arbeitete am 1914 eingeweihten und 1943 zerstörten St. Norbert Krankenhaus in Berlin-Schöneberg als dirigierender Arzt der Hals-Nasen-Ohrenabteilung und war in dieser Zeit auch wissenschaftlich äußerst aktiv. Kobrak wurde als jüdischem Arzt und Hochschullehrer 1933 aufgrund § 3 des Gesetzes zur Wiederherstellung des Berufsbeamtentums vom 7. April 1933 wegen „nicht arischer Abstammung“ die Lehrbefugnis entzogen. Nach Schikanen und Einschränkungen seiner ärztlichen Tätigkeit emigrierte er 1938 nach London, wurde englischer Staatsbürger und kehrte nach dem Krieg nach Berlin zurück. Er hielt auf dem ersten Nachkriegskongress der Gesellschaft Deutscher Hals-Nasen-Ohrenärzte 1949 in Karlsruhe einen bemerkenswerten Vortrag zur Physiologie des Labyrinthliquors. In der Folge erhielt Kobrak die Ehrenmitgliedschaft der Deutschen und der Österreichischen Fachgesellschaft (vgl. Schagen 2013 [74], Jauerneck 1955 [42]). Nach einem Wechsel des *Internationalen Zentralblatts für Ohrenheilkunde und Rhino-Laryngologie* zum Verlag Curt Kabitzsch, Leipzig, wurde ab 1936 der Titel in *Der Hals‑, Nasen- und Ohrenarzt. Teil 2 Übersichtsberichte u. Referate* geändert und dann 1943 das Erscheinen eingestellt. Der Titelverlauf, die Umbenennungen und die Herausgeber sind in Tab. 7 dargestellt. Seit 1909 hatte der Kabitzsch-Verlag parallel noch eine weitere HNO-Zeitschrift für Originalarbeiten im Programm, die *Zeitschrift für Laryngologie, Rhinologie und ihre Grenzgebiete*, welche bis heute als *Laryngo-Rhino-Otologie (LRO)* im Thieme-Verlag erscheint.

**Table 7:** 

1	***Internationales Centralblatt für Ohrenheilkunde***
	Leipzig: J. A. Barth, **1.** 1903 – **5.** 1907
	Hrsg. (1903–1907): Oskar Brieger, Breslau, Giuseppe Gradenigo, Turin
2	***Internationales Zentralblatt für Ohrenheilkunde***
	Leipzig: J. A. Barth, **6.** 1908 – **10.** 1912
	Hrsg. (1908–1912): Oskar Brieger, Breslau, Giuseppe Gradenigo, Turin
3	***Internationales Zentralblatt für Ohrenheilkunde und Rhino-Laryngologie***
	Leipzig: J. A. Barth, **11.** 1913 –** 20.** 1922
	Hrsg. (1913–1922): Oskar Brieger, Breslau, Giuseppe Gradenigo, Turin, Max Goerke, Breslau, Bernhard Heine, München, Jörgen Möller, Kopenhagen, Paul Stenger, Königsberg, Franz Kobrak, Berlin
4	***Internationales Zentralblatt für Ohrenheilkunde und Rhino-Laryngologie, Folia oto-laryngologica: Teil 2***
	Leipzig: Curt Kabitzsch, **21**. 1923 – **41.** 1935
	Hrsg. (1923–1935): Max Goerke, Breslau, Bernhard Heine, München, Franz Kobrak, Berlin, Jörgen Möller, Kopenhagen, Paul Stenger, Königsberg
5	***Der Hals-Nasen und Ohrenarzt, Teil 2, Übersichtsberichte und Referate***
	Leipzig: Curt Kabitzsch, **42.** 1936 – **52.** 1943, 1943; damit Ersch. eingest.
	Hrsg. (1936–1943): Wilhelm Berger, Königsberg, Alfred Brüggemann, Giessen, Helmut Loebell, Marburg/Münster, Hermann Marx, Würzburg, Jürgen Möller, Kopenhagen, Paul Stenger, Berlin, Alfred Seiffert, Kiel/Heidelberg

### Titelhistorie: *Verhandlungen der Gesellschaft Deutscher Hals‑, Nasen- und Ohrenärzte: Jahresversammlung*

#### Berlin; München: Bergmann 1. 1921 – 19. 1939, Leipzig: Kabitzsch [anfangs], und Folgende (Tab. 8; [16, 21])

**Figure 14:**
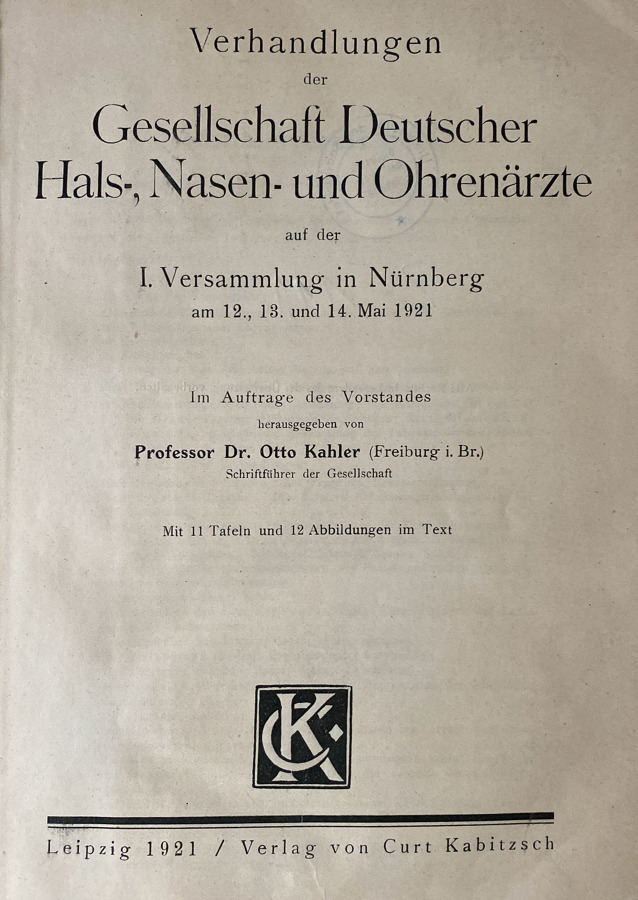

Bis 1914 wurden die Verhandlungen der „Deutschen Otologischen Gesellschaft“ und des „Vereins Deutscher Laryngologen“ in eigenen Zeitschriften publiziert (bei Fischer in Jena von 1895 bis 1914 bzw. bei Kabitzsch in Würzburg von 1909 bis 1914). Mit der Gründungsversammlung der Gesellschaft Deutscher Hals‑, Nasen- und Ohrenärzte 1921 in Nürnberg war auch die Frage des offiziellen Publikationsorgans der neuen wissenschaftlichen Fachgesellschaft zu klären. In der ersten Tagung der neuen Gesellschaft unter dem Vorsitz von Adolf Passow (1859–1926), Berlin, und Otto Kahler (1878–1946), Freiburg/Br., wurde eine Kommission beauftragt, die Verhandlungen mit den Verlagen zu führen [44]. Die *Verhandlungen der Gesellschaft Deutscher Hals‑, Nasen- und Ohrenärzte: Jahresversammlung *erschienen somit zunächst im Verlag Curt Kabitzsch in Leipzig und später bei Bergmann in Berlin und München. Die Abb. 14 zeigt das Titelblatt des ersten Bands der *Verhandlungen*. Herausgeber und zugleich Schriftleiter der Verhandlungen waren die jeweiligen Präsidenten und Schriftführer der Gesellschaft. Bei 92 Jahresversammlungen bis 2021 würde ihre Aufzählung den Rahmen dieser Publikation sprengen. Sie sind auf www.hno.org nachzulesen [15]. Die Ergebnisse der Jahresversammlungen wurden im weiteren Verlauf unter unterschiedlichen Titeln in unterschiedlicher Form bei verschiedenen Verlagen in Print- und später in Online-Versionen publiziert (Tab. 8, 9 und 10), aktuell als Supplementband in der LRO beim Thieme-Verlag, Stuttgart [16]. Den bedeutendsten Anteil nahmen die Referate und die Vorträge ein. Während die Referate bis heute i. d. R. eine Zusammenfassung aktueller Erkenntnisse darstellen, haben die Vorträge und Poster den Charakter von Originalarbeiten, welche aktuelle Ergebnisse von Studien publizieren. Die Form der Darstellung veränderte sich im Laufe der Jahrzehnte, sodass heute die Vorträge überwiegend in Kurzform als Abstracts oder als Poster nur noch online veröffentlicht werden. Erinnerlich ist die Publikation auch der Diskussionsbeiträge während der Jahresversammlungen bis in die 1990er-Jahre. In §§ 21–29 der ersten Vereinssatzung der Gesellschaft Deutscher Hals‑, Nasen- und Ohrenärzte war neben der Dauer und der Reihenfolge auch die Veröffentlichung der Diskussionsbeiträge explizit geregelt worden. Sitzungsberichte und Protokolle der Mitgliederversammlungen der Gesellschaft wurden anfangs in den *Verhandlungen* publiziert. Seit 1976 werden diese in den *HNO-Informationen* beim Demeter Verlag, Gräfelfing, Balingen und Stuttgart, und aktuell beim Deutschen Ärzteverlag, Köln, veröffentlicht. Der Titelverlauf ist in Tab. 8 dargestellt.

**Table 8:** 

1	***Verhandlungen der Gesellschaft Deutscher Hals‑, Nasen- und Ohrenärzte: Jahresversammlung***
	Berlin; München: Bergmann **1.** 1921 – **19.** 1939, Leipzig: Kabitzsch [anfangs]
	Hrsg. (1921–1931): Präsident und Schriftführer der Deutschen Gesellschaft der Hals‑, Nasen‑, Ohrenärzte
2	***Verhandlungen der Deutschen Gesellschaft der Hals‑, Nasen‑, Ohrenärzte: Jahresversammlung***
	Berlin [u. a.]: Springer **20.** 1949 – **39.** 1968
	Hrsg. (1949–1968): Präsident und Schriftführer der Deutschen Gesellschaft der Hals‑, Nasen‑, Ohrenärzte
3	***Verhandlungen der Deutschen Gesellschaft für Hals-Nasen-Ohren-Heilkunde, Kopf- und Hals-Chirurgie: auf der … Jahresversammlung***
	Berlin; Heidelberg [u. a.]: Springer **40.** 1969 – **53.** 1982
	Hrsg. (1969–1982): Präsident und Schriftführer der Deutschen Gesellschaft für Hals-Nasen-Ohren-Heilkunde, Kopf- und Hals-Chirurgie
4	***Verhandlungsbericht …/Deutsche Gesellschaft für Hals-Nasen-Ohren-Heilkunde, Kopf- und Halschirurgie***
	Stuttgart; New York: Thieme, Berlin; Heidelberg [u. a.]: Springer [anfangs], 1983–1996,1; 1997,1; 1999–2015, 1996,2; 1997,2 – 1998 nicht ersch.; unregelmäßig, ist Beilage zu: 1993–1996,1 Suppl. zu: European archives of oto-rhino-laryngology, weitere Titelhinweise: 1990,1 – 1996,1 zugl. Bd. von: Deutsche Gesellschaft für Hals-Nasen-Ohren-Heilkunde, Kopf- und Hals-Chirurgie: Jahresversammlung der Deutschen Gesellschaft für Hals-Nasen-Ohren-Heilkunde, Kopf- und Hals-Chirurgie, Einzelne Bd. zugl. Bd. von: Deutsche Gesellschaft für Hals-Nasen-Ohren-Heilkunde, Kopf- und Hals-Chirurgie: Referate, 2001 = 80, Suppl. 1; 2010 = 89, Suppl. von: Laryngo-Rhino-Otologie, 1983–1992 zugl. alle Bd. von: [Archives of oto-rhino-laryngology/Supplement]
	Hrsg. (1983–2015): Präsident und Schriftführer der Deutschen Gesellschaft für Hals-Nasen-Ohren-Heilkunde, Kopf- und Hals-Chirurgie

**Table 9:** 

***GMS Current topics in otorhinolaryngology—head and neck surgery: CTO/Deutsche Gesellschaft für Hals‑, Nasen‑, Ohrenheilkunde, Kopf- und Halschirurgie e. V.***
Düsseldorf: gms Nachgewiesen **3**. 2004 – volume **16** (2017); damit Erscheinen eingestellt, https://www.egms.de/dynamic/de/journals/
Hrsg. (2004–2017): Hans-Jürgen Schultz-Coulon, Neuss, Eggert Beleites, Jena, Karl Hörmann, Mannheim, Alexander Berghaus, München, Friedrich Bootz, Bonn, Hans-Wilhelm Pau, Rostock, Gerhard Rettinger, Ulm, Roland Laszig, Freiburg/Br., Norbert Stasche, Kaiserslautern, Heinrich Iro, Erlangen, Thomas Deitmer, Dortmund, Werner Hosemann, Greifswald, Jochen A. Werner, Essen, Dirk Eßer, Erfurt

**Table 10:** 

***GMS current posters in otorhinolaryngology, head and neck surgery: CPO; …*** ***Jahresversammlung der Deutschen Gesellschaft für Hals-Nasen-Ohren-Heilkunde, Kopf- und Hals-Chirurgie***
Düsseldorf: gms **1**. 2005 – volume **13** (2017); damit Erscheinen eingestellt, https://www.egms.de/dynamic/en/journals/
Hrsg. (2004–2017): Hans-Jürgen Schultz-Coulon, Neuss, Eggert Beleites, Jena, Karl Hörmann, Mannheim, Alexander Berghaus, München, Friedrich Bootz, Bonn, Hans-Wilhelm Pau, Rostock, Gerhard Rettinger, Ulm, Roland Laszig, Freiburg/Br., Norbert Stasche, Kaiserslautern, Heinrich Iro, Erlangen, Thomas Deitmer, Dortmund, Werner Hosemann, Greifswald, Jochen A. Werner, Essen, Dirk Eßer, Erfurt

### Titelhistorie: *GMS Current topics in otorhinolaryngology—head and neck surgery: CTO*

#### *Deutsche Gesellschaft für Hals‑, Nasen‑, Ohrenheilkunde, Kopf- und Halschirurgie e. V., Düsseldorf: gms Nachgewiesen 3. 2004 – volume 16 (2017); damit Erscheinen eingestellt* und *GMS current posters in otorhinolaryngology, head and neck surgery: CPO; … Jahresversammlung der Deutschen Gesellschaft für Hals-Nasen-Ohren-Heilkunde, Kopf- und Hals-Chirurgie, *Düsseldorf: gms 1. 2005 – volume 13 (2017); damit Erscheinen eingestellt, [22, 24]

Es waren vornehmlich ökonomische Gründe und die Förderung von Open-Access-Publikationen durch den deutschen Staat, welche das Präsidium der Deutschen Gesellschaft für Hals-Nasen-Ohren-Heilkunde, Kopf- und Hals-Chirurgie veranlasste, die englischen Übersetzungen der Referate der Jahresversammlungen, die deutschsprachigen Abstracts und die Poster ab 2004 in einer neu gegründeten elektronischen Fachzeitschrift zu publizieren. Das Portal „GMS – German Medical Science“ ist ein Zusammenschluss der Arbeitsgemeinschaft der Wissenschaftlichen Medizinischen Fachgesellschaften (AWMF), des Deutschen Instituts für Medizinische Dokumentation und Information (DIMDI) und der Deutschen Zentralbibliothek für Medizin (ZB MED). Gemäß den Open-Access-Bedingungen besteht eine Creative-Common-Lizenz, welche eine freie Verfügbarkeit der durch staatliche Förderung erlangten Forschungsergebnisse gewährleistet. Die elektronischen Fachzeitschriften GMS-CTO und GMS-CPO bestanden bis zum Band 16 bzw. 13 und wurden 2017 wieder eingestellt. Seit der 89. Jahresversammlung der Deutschen Gesellschaft für Hals-Nasen-Ohren-Heilkunde, Kopf- und Hals-Chirurgie 2018 werden die deutsch- und englischsprachigen Referate in der Zeitschrift LRO Supplement 1, die deutsch- und englischsprachigen Abstracts und die Poster in der LRO Supplement 2 publiziert und sind über eine Verlinkung auf der Webseite der Fachgesellschaft auch online frei abrufbar [16]. Titelverlauf und Herausgeber sind in den Tab. 9 und 10 dargestellt.

## Beispiele für einen Paradigmenwechsel in der praktischen und wissenschaftlichen HNO-Heilkunde

Selten sind Einzelpublikationen dazu geeignet, einen nachhaltigen Einfluss auf Krankheitsverständnis, Diagnostik oder Therapie zu nehmen. Solche Umbrüche im wissenschaftlichen Verständnis werden i. d. R. auch erst im Nachhinein wahrgenommen. So kann man für das Fachgebiet der HNO im historischen Rückblick Leistungen einzelner Wissenschaftler und Kliniker identifizieren, welche zu Paradigmenwechseln und in ihrer Zeit zu erheblichen Kontroversen geführt haben. Einige wenige werden hier anhand von Publikationen in deutschsprachigen HNO-Zeitschriften gesondert erwähnt. Es ist allerdings nicht möglich, alle bedeutenden Publikationen der letzten 200 Jahre aufzuzählen und zu würdigen. Zu viele Autoren bleiben unberücksichtigt, die folgende Auswahl ist deshalb auch stark subjektiv.

Hermann Schwartze veröffentlichte 1873 die Originalarbeit „Über die künstliche Eröffnung des Warzenfortsatzes“ im *Archiv für Ohrenheilkunde*. Er reaktivierte eine in über 100 Jahren nur vereinzelt durchgeführte Operationsmethode, welche durch falsche Indikationsstellung und ungenügend wissenschaftlich begründete Operationstechnik in Misskredit geraten war. Schwartze hatte sich im Jahr 1863 in Halle an der Saale für das Fach Ohrenheilkunde habilitiert. Dies in einer Zeit in der „… schwere Vorurtheile, welche, durch frühere Irrthümer hervorgerufen, auf der Ohrenheilkunde lasteten“ [49]. Nach Jahren anatomischer, klinischer und pathologischer Studien veröffentlichte er zusammen mit seinem Assistenten Adolf Eysell (1846–1934) 1873 seine Arbeiten „Über die künstliche Eröffnung des Warzenfortsatzes“ [83–85]. Eysell war bis 1875 Assistent an der Ohrenklinik in Halle an der Saale, bevor er ab 1876 in Kassel als Hals-Nasen-Ohrenarzt praktizierte und ab da nur noch entomologische Abhandlungen publizierte [99]. Schwartze und Eysell definierten nicht nur erstmals eine wissenschaftlich begründete Indikation, sondern beschrieben auch die Operationstechnik mit Hammer und Meißel zur Mastoideröffnung. Schwartze lehnte die unkritische, blinde Trepanation seiner Vorgänger ab und konnte mit seinen ebenfalls veröffentlichten klinischen Erfolgen seine Kritiker widerlegen. Schwartze gilt noch heute als Vater der modernen Mastoidchirurgie. Von Schwartzes ersten 100 beschriebenen operativen Fällen starben 20, und 74 Fälle wurden geheilt. „In keinem Falle war der letale Ausgang mit Bestimmtheit als directe Folge der Operation zu betrachten“ [82]. Die Indikationen gibt Schwartze mit subperiostalen Abszessen (62 Fälle), akuter Entzündung am Warzenfortsatz (17 Fälle) und Eiterretention im Mittelohr (13 Fälle) an. Bemerkenswert ist auch, dass bei 9 von 39 geheilten Fällen der zweiten Serie eine völlige Normalisierung des Hörvermögens erreicht werden konnte [82]. Diese zu damaliger Zeit herausragenden und bemerkenswerten Ergebnisse einer Eröffnung des Warzenfortsatzes, bei den meisten Patienten in einer lebensbedrohenden Situation ausgeführt, führten sehr bald zu einer weiten Akzeptanz und Verbreitung der Mastoidektomie. Interessant an der Publikation der Auswertung der ersten 100 Fälle ist nicht nur die Analyse der Einzelfälle, sondern auch die kritische Auseinandersetzung mit den Komplikationen [82]. Wenn auch retrospektiv, so stellt diese Veröffentlichung von Schwartze eine frühe Form einer klinischen Studie dar. In den ersten Jahrzehnten wurden dagegen im *Archiv für Ohrenheilkunde* im wesentlichen Kasuistiken, Originalarbeiten mit überwiegend beschreibendem Charakter, Patientenstatistiken aus Ohrenkliniken, Conference Proceedings und Buchbesprechungen publiziert [63].

Im Jahr 1944 hatte in Aix en Provence ein deutscher HNO-Arzt als „Prisoner of War“ in amerikanischer Kriegsgefangenschaft einen regen wissenschaftlichen Austausch mit amerikanischen Militärärzten. Sein Name war Horst Wullstein (1906–1987). Er nahm dabei die Gelegenheit wahr, die von Dr. Weiss aus New York City mitgebrachte Literatur von Lempert (1891–1968) und Shambaugh (1903–1999) zu den Ergebnissen der zu dieser Zeit in den USA zunehmend verbreiteten Fensterungsoperationen zu studieren [107]. Basierend auf den Erfolgen dieser Fensterungsoperationen bei Otosklerose kam der Gedanke auf, nach der operativen Sanierung von chronischen Mittelohreiterungen auch das Gehör mit derselben Technik zu verbessern. Nachdem Horst Wullstein, Anfang der 1950er-Jahre noch Chefarzt in Siegen, zahlreiche Fensterungsoperationen selbst ausgeführt hatte, unternahm er den Versuch, die Prinzipien der Schallprotektion, der Columellisation und des Verschlusses des Trommelfells mit Spalthauttransplantaten auf die Rekonstruktion des Mittelohrs anzuwenden. Bei 1000 durchgeführten Fensterungsoperationen hatte er bei knapp der Hälfte der Fälle eine gestörte Warzenfortsatzpneumatisation oder direkte entzündliche Mittelohrveränderungen gesehen. In seiner Originalarbeit „Funktionelle Operationen im Mittelohr mit Hilfe des freien Spaltlappen-Transplantates“ [105], veröffentlicht 1952 im *Archiv für Ohren‑, Nasen- und Kehlkopfheilkunde*, beschrieb er erstmals das Konzept einer hörverbessernden Operation. Den Terminus „Tympanoplastik“ und die Beschreibung der Tympanoplastiktypen sollte Wullstein erst in seinem Buchbeitrag in der Operationslehre von Uffenorde einführen [106]. Die Originalarbeit im *Archiv für Ohren‑, Nasen- und Kehlkopfheilkunde* beschrieb jedoch schon ausführlich die verschiedenen Operationstechniken, welche durch zahlreiche instruktive Zeichnungen unterstützt wurden. Von mehr als 10 Patienten wurden der klinische Verlauf und die Audiogramme dargestellt. Diese erstmals beschriebenen Grundsätze der hörverbessernden Operationen bei chronischen Mittelohreiterungen führten dazu, dass die Würzburger Klinik in den Folgejahren von zahlreichen Ohrchirurgen aus aller Welt besucht wurde und die Tympanoplastik mit ihrer Einteilung in verschiedene Typen nach Wullstein noch heute weltweit anerkannt ist.

Im Jahr 1964, d. h. 6 Jahre nach Beginn der Facharztausbildung in der HNO bei Seiferth in Köln, veröffentlichte Oskar Kleinsasser (1929–2001) erstmals sein Konzept einer „Mikrochirurgie im Kehlkopf“ [50]. Kleinsasser, ursprünglich ausgebildet als Pathologe, hatte sich 2 Jahre zuvor zur Frühdiagnose des Kehlkopfkarzinoms habilitiert. In seiner Originalarbeit zur Mikrochirurgie beschreibt er nicht nur die speziellen Probleme der Anpassung eines Mikroskops an ein Schwebelaryngoskop, sondern auch die technische Realisierung in Zusammenarbeit mit der Industrie. Auch verband ihn ein jahrelanger Austausch mit Geza Jako aus Boston, MA, bei der Weiterentwicklung eines Lupenlaryngoskops zum Kehlkopfoperationsmikroskop [50]. Zusätzlich war die Entwicklung eines gänzlich neuen, praxistauglichen Instrumentariums und von Laryngoskoprohren notwendig. Bis heute werden die von Kleinsasser für die Mikrochirurgie im Kehlkopf entwickelten Instrumente eingesetzt und tragen seinen Namen. Ein besonderes, in der Publikation erwähntes Problem war der anfangs zu geringe Arbeitsabstand zwischen binokularem Operationsmikroskop und Laryngoskop. Erst durch die Bereitstellung einer 400-mm-Optik durch den Mikroskophersteller konnte ein genügend großer Arbeitsabstand gewährleistet werden [50]. Bis heute geht das Standardsetting der Mikrolaryngoskopie mit den langen, speziellen Instrumenten, dem Operationsmikroskop mit 400 mm Objektivbrennweite und den speziellen Rohrlaryngoskopen auf die grundlegenden Arbeiten von Kleinsasser in den 1960er-Jahren zurück. Schon etwa 10 Jahre vorher hatte Rosemarie Albrecht aus Erfurt, inspiriert durch die Erfolge ihrer Kollegen in der Gynäkologie bei der Früherkennung von malignen Läsionen an der Cervix uteri, ein geliehenes Vergrößerungskolposkop im Kehlkopf eingesetzt [1]. Aufgrund der erfolgreichen Zusammenarbeit mit Mikroskop- und Instrumentenherstellern und der innovativen Entwicklungen sowohl von Geza Jako als auch von Oskar Kleinsasser ist die Mikrolaryngoskopie heute ein weltweit akzeptiertes Verfahren zur endolaryngealen Diagnostik und Chirurgie.

Walter Messerklinger (1920–2001), von 1959–1990 Leiter der Grazer Universitäts-HNO-Klinik, gilt noch heute als der Pionier der endoskopischen Chirurgie der chronischen Sinusitis. Durch seine grundlegenden Arbeiten „Über die Drainage der menschlichen Nasennebenhöhlen unter normalen und pathologischen Bedingungen“ [59] im Jahr 1966 stieß er eine bis heute dauernde Entwicklung an. Dies führte in den 1980er- und 1990er-Jahren zu einem Wandel der operativen Behandlung der chronischen Rhinosinusitis weg von der radikalen, schleimhautresezierenden, hin zu einer funktionellen, schleimhautschonenden Chirurgie. Die in der *Monatsschrift Ohrenheilkunde, Laryngorhinologie* publizierte Arbeit beschreibt nicht nur detailliert den normalen Transport des markierten Sekrets unter dem Operationsmikroskop in den Nasennebenhöhlen an der menschlichen Leiche, sondern auch die Sekretdrainage der kranken Sinusschleimhaut intra operationem. So konnte er den damals herrschenden Streit über die optimale operative Anlage eines Kieferhöhlenfensters im unteren oder mittleren Nasengang wegen des nachzuweisenden Zilientransports zum natürlichen Ostium der Kieferhöhle im mittleren Nasengang entscheiden: „Bei operierten Kieferhöhlen wird … der neue Ausführungsgang in den unteren Nasengang meist umgangen und das natürliche Ostium benutzt“ [59]. Es dauerte dann 20 Jahre, bis Messerklinger und sein Schüler Stammberger (1946–2018) das auf diesen Zilientransportuntersuchungen basierende Konzept einer neuen Pathophysiologie der chronischen Rhinosinusitis [60] und der darauf fußenden endoskopischen Nasennebenhöhlenchirurgie publizieren konnten [90, 91]. Die Veröffentlichungen erfolgten Mitte der 1980er-Jahre in der *Laryngologie, Rhinologie, Otologie: Zeitschrift für Hals-Nasen-Ohrenheilkunde*, Nachfolger der Monatsschrift und Publikationsorgan auch der Österreichischen Oto-Laryngologischen Gesellschaft und der Wiener Gesellschaft der Hals‑, Nasen‑, Ohren-Ärzte [18].

Hauptvorträge auf Kongressen haben selten einen absoluten Neuigkeitswert und werden noch seltener kontrovers diskutiert, fassen sie doch i. Allg. nur das aktuelle Wissen zu einem Thema zusammen. Bemerkenswert ist dennoch das Ereignis eines Hauptvortrags im Jahr 1987. Wolfgang Steiner (1942–), frischberufener HNO-Lehrstuhlinhaber und Klinikdirektor in Göttingen, erhielt 1987 vom damaligen Präsidenten Klaus Terrahe (1935–) den Auftrag, für die Jahresversammlung der Deutschen Gesellschaft für Hals-Nasen-Ohren-Heilkunde, Kopf- und Hals-Chirurgie in Bad Neuenahr einen Hauptvortrag zum Thema „Laserchirurgie im HNO-Bereich (Laserchirurgie zur Behandlung von malignen Tumoren des oberen Aerodigestivtraktes)“ zu halten. Steiner hatte seit 1979 in Erlangen begonnen, maligne Tumoren des oberen Aerodigestivtrakts laserchirurgisch zu behandeln und konnte zu diesem Zeitpunkt über Erfahrungen an 900 Patienten berichten. Der Hauptvortrag ist im Supplementband des *Archivs für Otorhinolaryngologie* veröffentlicht worden [93]. Steiner bezeichnete die Publikation selbst als „persönlichen Erfahrungsbericht“, beschreibt hier aber nicht nur die „Möglichkeiten und Grenzen palliativ symptomatischer sowie kurativer Mono- und Kombinationstherapie“, sondern erstmals die Grundzüge der transoralen Lasermikrochirurgie für die Behandlung von malignen Tumoren des oberen Aerodigestivtrakts. Für die einzelnen Tumorregionen und -stadien werden Vorgehensweise, Indikationen und Kontraindikationen detailliert beschrieben. Mit dem Verzicht auf die traditionelle Blockresektion und Defektdeckung bei fortgeschrittenen Tumoren steht sein laserchirurgisches Konzept mit den bis dahin festgeschriebenen Vorgehensweisen im deutlichen Widerspruch. Primärtumor und Hals werden zeitlich getrennt operiert, das regionale Lymphabflussgebiet wird funktionserhaltend „häufig sogar regional begrenzt“ operiert, der Primärtumor wird mikrochirurgisch reseziert und auf eine aufwendige Defektdeckung kann verzichtet werden. Dieser Paradigmenwechsel in der operativen Behandlung, vor allen Dingen von fortgeschrittenen Kopf-Hals-Tumoren, konnte 1987 natürlich nicht unwidersprochen bleiben. Die Publikation des Hauptvortrags im *Archiv für Otorhinolaryngologie *beinhaltet auch die damals noch übliche schriftliche Wiedergabe der Diskussion im Vortragssaal. Insbesondere Oskar Kleinsasser, Ordinarius in Marburg, erhob heftige Kritik an der Indikation zur endoskopischen Resektion fortgeschrittener Stimmlippenkarzinome, indem er die bessere Übersicht bei einem externen Zugangsweg und die dabei mögliche Rekonstruktion der resezierten Stimmlippe anführt. Kleinsasser ergänzte zudem: „bei Epiglottiskarzinomen und Hypopharynxkarzinomen gehen unsere Meinungen diametral auseinander“ [93]. Insbesondere die laserchirurgische Tumorverkleinerung mit anschließender Bestrahlung sah er kritisch, und die Wundheilung erinnere ihn manchmal an einen „ausgebrannten Krater“ [93]. Kleinsasser kritisierte das Fehlen einer brauchbaren Statistik heftig, weshalb die Ausführungen „wissenschaftlich gesehen nur einen anekdotischen Wert“ [93] hätten. Die kontroversen Diskussionen setzten sich auf den folgenden Jahresversammlungen fort. Es ist der Hartnäckigkeit und Weitsicht von Wolfgang Steiner zu verdanken, dass sich die von ihm maßgeblich vorangetriebene transorale Lasermikrochirurgie (TLM) weltweit etabliert hat und auch neben der transoralen roboterassistierten Chirurgie (Transoral Robotic Surgery, TORS) zur Behandlung von Kopf-Hals-Tumoren bestehen kann.

## Die großen Medizin-Verlage

### Springer

Julius Springer (1817–1877) ist der Stammvater der weitverzweigten Springer-Familie, die neben Buchhändlern und Verlegern auch Ingenieure, Juristen, Künstler, Galeristen und Wissenschaftler hervorgebracht hat. Die Geschichte des Springer-Verlags beginnt im Jahr 1832, als der in Berlin geborene Julius Springer im Alter von 15 Jahren eine Lehre in der Berliner Enslin’schen Buchhandlung antrat. Nach seiner Lehrzeit schlossen sich 4 buchhändlerische Wanderjahre an, die ihn zunächst nach Zürich führten, wo er u. a. Georg Büchner kennen lernte. Nach 2 Jahren in der Schweiz zog er 1838 weiter und wurde Gehilfe des Stuttgarter Buchhändlers Neff, wo erste Pläne zur Gründung einer eigenen Buchhandlung reiften. Nach einer weiteren Stellung in der Pariser Buchhandlung Brockhaus Avenarius kehrte er 1840 nach Berlin zurück.

Der 25-jährige Springer erhielt 1842 eine Konzession zum Betrieb eines Buchhandels in Berlin, in dem es zu jener Zeit bereits etwa 100 buchhändlerische Unternehmen gab. Alle größeren Buchhandlungen in Deutschland hatten seinerzeit einen Kommissionär in Leipzig, der die Aufträge der Buchhändler bei den Verlagen einreichte und im Anschluss die bestellte Ware ausliefern ließ. Bei der zunehmenden Bedeutung von Berlin als Verlagsstadt war es für norddeutsche Sortimenter aber unwirtschaftlich und zeitraubend, sich auch die Berliner Produktion über Leipzig kommen zu lassen. So wurde Julius Springer zum Kommissionär und stand 1845 mit 20 auswärtigen Kommittenten neben seiner Lehrfirma Enslin in Berlin schon an erster Stelle.

Der „Verlag von Julius Springer“ publizierte zunächst überwiegend politische Karikaturen und Schriften im Sinne des Vormärz. Seine politischen Aussagen, in denen er u. a. die willkürliche Zensur durch die preußische Staatsgewalt beklagte, führten auch zu juristischen Verwicklungen. Springer wurde u. a. in seiner Buchhandlung verhaftet und für 8 Tage inhaftiert, weil er oppositionelle Schriften veröffentlichte.

Nach dem Verkauf der Buchhandlung 1858 widmete sich Springer ausschließlich dem Verlagsgeschäft. Allmählich rückte neben den politischen Schriften auch spezialisierte Fachliteratur aus dem Bereich der Naturwissenschaften und Technik in den Fokus des Verlags. Im Frühjahr 1871 nahm der Verlagsgründer seinen ältesten Sohn Ferdinand (1846–1906) in den Verlag auf und machte ihn wenig später zum Teilhaber. Hiernach nahm die Titelproduktion rasch zu und stieg von 161 Produktionen im Jahr 1871 innerhalb weniger Jahre auf 200 Titel an. Nach dem Tod des Firmengründers Julius Springer im Jahr 1877 nahm Ferdinand Springer seinen jüngeren Bruder Fritz (1850–1944) in den Verlag auf. In den 1880er-Jahren begann der erste große Aufschwung des Verlags durch die Gründung oder Übernahme zahlreicher Zeitschriften. Diese Veröffentlichungen, die z. T. noch heute verlegt werden, waren Schrittmacher für die Entwicklung des Verlags in den folgenden Jahrzehnten und haben zu seiner wirtschaftlichen Stabilität einen entscheidenden Beitrag geleistet. Im Jahr 1877 hatte der Verlag 4 Mitarbeiter, 1906 waren es schon 65.

Als Springers erste Medizinzeitschrift erschienen 1887 die *Therapeutischen Monatshefte*, deren Autoren renommierte Wissenschaftler wie z. B. der spätere Nobelpreisträger Paul Ehrlich waren. Ein Jahr nach der Gründung hatten die Monatshefte schon über 4000 Abonnenten. Bis 1887 wurden 12 medizinische Publikationen veröffentlicht, 10 Jahre später waren es schon fast 100. Ferdinand und Fritz Springer nahmen frühzeitig ihre Söhne Ferdinand d. J. (1881–1965) und Julius d. J. (1880–1968) in den Verlag auf, die diesen ab 1907 leiteten (vgl. [73]).

Nach einer langanhaltenden Phase stetigen Wachstums kam es 1905 infolge einer Wirtschaftsschwäche vorübergehend zu einem Umsatzrückgang um etwa 20 % in allen Verlagsbereichen, auch die Technik und Medizin waren davon betroffen. Schon 1910 jedoch überschritt die Jahresproduktion zum ersten Mal 200 Titel, weitere 3 Jahre später produzierte der Verlag schon 310 Titel, davon 60 Zeitschriften. Im Jahr 1914 lag der Springer-Verlag mit 379 aufgelegten Werken nach dem Leipziger Teubner-Verlag bereits an zweiter Stelle im Deutschen Reich. In dieser Zeit beginnt auch die Internationalisierung der Wissenschaft: Das Zentrum der wissenschaftlichen Welt sind nicht mehr Deutschland und Europa, sondern die USA; das Englische entwickelt sich zur unangefochtenen Lingua franca der Wissenschaft [86].

Kurz nach Ausbruch des 1. Weltkriegs im August 1914 kam es zu einem dramatischen Umsatzeinbruch im deutschen Verlagswesen. „Es wird nichts verkauft“ umschrieb Fritz Springer die damalige Situation (vgl. Sarkowski 1992, S. 226 ff. [73]). Da der Verkauf so stark zurückgegangen war, fehlten zudem auch die Mittel, um neue Projekte zu finanzieren. Der Springer-Verlag war besonders von der Krise betroffen, da die beiden Verlagsköpfe Ferdinand und Julius Springer als Reserveoffiziere schon Anfang August 1914 an die Front gekommen waren. So wurden 1915 nur noch 108 Titel produziert. Vom Produktionsrückgang war der Fachbereich Medizin besonders betroffen, da viele Ärzte beim Militär waren, und die daheimgebliebenen wegen Überlastung ihre schriftstellerische Arbeit nicht fortführen konnten. Ein Beispiel hierfür ist das „Handbuch der inneren Medizin“: Der Beitrag „Leber und Gallenwege. Pankreas“ war zu Kriegsbeginn druckreif, die übrigen Beiträge mussten verschoben werden. So erschien zunächst nur der bereits fertiggestellte Beitrag mit einem Umfang von 186 Seiten, der Rest mit über 1700 Seiten kam erst 1918 heraus [73].

Im Jahr 1918 gelang die wichtige Übernahme des Medizinverlags J. F. Bergmann, der zu diesem Zeitpunkt im Bereich Medizin bedeutender als der Springer-Verlag war. Unter den dazu gewonnenen Titeln befand sich auch die *Zeitschrift für Ohrenheilkunde*, welche 1878 aus dem von Knapp, Mauthner und Moos herausgegebenen *Archiv für Augen- und Ohrenheilkunde* hervorgegangen war. Die Zahl der von Springer von 1918 bis zum Ende der Inflation verlegten Zeitschriften nahm um mehr als das Doppelte zu. Dieser Zuwachs resultierte zu einem erheblichen Teil aus dem Erwerb der Verlage J. F. Bergmann (1918) mit 10 Titeln und A. Hirschwald (1921) mit 12 Titeln. Hinzu kamen 18 Zeitschriften aus anderen Verlagen sowie 12 Neugründungen bzw. Umwandlungen bisheriger Buchserien in Zeitschriften.

Die extreme Inflation der Nachkriegszeit brachte viele deutsche Verlage in existenzielle Not. Als einem der wenigen Verlage gelang es Springer, selbst in dieser wirtschaftlich schwierigen Zeit zu expandieren. So wurde 1921 die in Berlin ansässige Hirschwald’sche Buchhandlung übernommen nebst der Zeitschrift *Archiv für Laryngologie und Rhinologie*. Bei der Übernahme durch Springer hatte diese Buchhandlung nur 12 Angestellte. Innerhalb von 10 Jahren erhöhte sie ihren Personalstand auf 154 Mitarbeiter, womit Hirschwald unter dem Dach von Springer zum Ende der Weimarer Republik Deutschlands größte wissenschaftliche Buchhandlung war.

Mit dem Ankauf des bereits im Jahr 1730 gegründeten Leipziger Verlags F. C. W. Vogel erfolgte 1931 eine weitere wichtige Expansion des Springer-Verlags. Bedeutend war insbesondere dessen übernommenes Zeitschriftenportfolio, u. a. das 1864 gegründete und 1915 in *Archiv für Hals-Nasen- und Ohrenheilkunde* umbenannte *Archiv für Ohrenheilkunde.*

Wenige Wochen nach Ernennung Adolf Hitlers zum Reichskanzler durch den Reichspräsidenten Paul von Hindenburg am 30. Januar 1933 wurde mit dem „Gesetz zur Wiederherstellung des Berufsbeamtentums“ eine Welle von Entlassungen nicht arischer und insbesondere jüdischstämmiger Beamten und Angestellten vollzogen. So verloren auch Professoren und Institutsdirektoren ihre Lehrbefugnis und in der Folge die Möglichkeit, sich publizistisch in den verschiedenen Verlagen zu betätigen. Innerhalb kurzer Zeit wurden über 2400 Wissenschaftler ihrer Stelle enthoben. Der Anteil jüdischer Autoren und Herausgeber von Buchserien und Zeitschriften war in den Naturwissenschaften und damit gerade im Springer-Programm relativ hoch. Zwischen 1933 und 1938 mussten mehr als 50 jüdische Zeitschriftenherausgeber und -redakteure beim Springer-Verlag im Rahmen der nationalsozialistischen Gleichschaltung der wissenschaftlichen Fachpresse ausscheiden (vgl. Sarkowski [73]).

Julius Springer d. J. hatte jüdische Großeltern und musste infolge des „Reichsbürger-Gesetzes“ 1935 den Verlag verlassen. Im Jahr 1941 wurde eine Verordnung erlassen, dass Unternehmen mit jüdischen Namen umzufirmieren seien, so musste sich der „Verlag von Julius Springer“, wie er seit seiner Gründung hieß, in „Springer-Verlag“ umbenennen. Auch die 1921 erworbene Hirschwald’sche Buchhandlung musste umbenannt werden und firmierte ab 1941 unter dem Namen „Lange Springer“. Schließlich ordnete 1942 die Reichsschrifttumskammer an, dass nach Julius Springer d. J. nun auch Ferdinand Springer aus seinen Firmen ausscheiden müsse. Alleinige Gesellschafter wurden die Brüder Tönjes Lange (1889–1961) und Otto Lange (1887–1967), seinerzeit Geschäftsführer der Wiener Dependance des Verlags. Fritz Springer nahm sich 1944 das Leben, um einer Deportation zu entkommen. Sein Bruder Ernst Springer, bis 1936 Jurist in der Reichsschuldenverwaltung, starb 1944 im Konzentrationslager Theresienstadt (vgl. Sarkowski 1992 [73]). Wirtschaftlich hatte der Verlag zunächst deutlich weniger zu leiden als während des 1. Weltkriegs. Die Inlandsumsätze stiegen seit der Machtübernahme der Nationalsozialistischen Deutschen Arbeiterpartei (NSDAP) kontinuierlich an, allerdings brach das Auslandsgeschäft drastisch ein, und die zunehmenden alliierten Luftangriffe auf Verlagsgebäude und Druckereien brachten das Verlagsgeschäft ab 1943 sukzessive zum Erliegen.

In den Nachkriegsjahren bestand zunächst ein Zwang zur Lizenzierung bei den amerikanischen Besatzungsbehörden, die Springer für die Wiederaufnahme verschiedener wichtiger Zeitschriften zunächst allerdings verweigert wurde. Schließlich wurde eine Reihe bedeutender Zeitschriften des Verlags wieder aufgelegt, die zwischenzeitlich eingestellt werden mussten. Die *HNO* erschien erstmals 1947. Dann kam 1948 das *Zentralblatt für Hals‑, Nasen- und Ohrenheilkunde* hinzu. Das *Archiv für Ohren‑, Nasen- und Kehlkopfheilkunde*, vereinigt mit der *Zeitschrift für Hals‑, Nasen- und Ohrenheilkunde*, erschien 1949. Die Erneuerung der 59 bestehenden und die Gründung weiterer 8 Zeitschriften zwischen 1946 und 1950 kennzeichnen die Produktivität jener Jahre. Ende 1950 belief sich die Mitarbeiterzahl in Berlin bereits auf 186 und in Heidelberg auf 57. Vom ersten Firmensitz im Berliner Westen zog das Unternehmen 1958 an den heutigen Standort am Heidelberger Platz um. Aufgrund der politischen Lage und der Isolierung des Berlins der Nachkriegszeit entschied sich Ferdinand Springer d. J. zur Dezentralisierung des Verlagsgeschäfts und eröffnete in Heidelberg und Göttingen Verlagszweige.

Auch der in München ansässige und 1918 von Springer übernommene J. F. Bergmann-Verlag erhielt 1948 eine Lizenz der amerikanischen Militärregierung. Die medizinischen Bestände des Verlags J. F. Lehmanns wurden 1977 übernommen, in dessen Sitz in München der Verlag J. F. Bergmann übersiedelte. Im Rahmen des Konzentrationsprozesses wurde 1989 J. F. Bergmann in den Springer-Verlag integriert.

Seine erste Tochterfirma außerhalb des deutschen Sprachraums gründete der Springer-Verlag im Rahmen der Internationalisierung 1964 in New York. Zwischen 1970 und 1990 entstanden weitere Tochterfirmen in London, Tokio, Paris, Hongkong, Barcelona und Budapest. Vor allem Asien etablierte sich als Markt, so begann 1978 die Übersetzung und Publikation chinesischer Wissenschaftswerke ins Englische. Die Übernahme weiterer Verlage wie J. F. Lehmanns, Birkhäuser und Steinkopff beschleunigte den wirtschaftlichen Erfolg. Erstmals mehr als 1000 Mitarbeiter hatte der Verlag 1988.

Ein medizinisch-biologisches Zentralblattsystem hatte Springer schon 1911 eingeführt, welches in Heidelberg u. a. die medizinische Literatur auswertete. Zum Zeitpunkt der umfangreichsten Recherchen im Jahr 1978 gab es 18 verschiedene Referateorgane mit 80 Redakteuren und Mitarbeitern. Diese werteten 2600 Zeitschriften von 5600 Referenten in 40 Staaten aus. Aus dem Bereich der HNO-Heilkunde bestand ein derartiges Medium mit dem *Zentralblatt für Hals‑, Nasen- und Ohrenheilkunde sowie deren Grenzgebiete* bereits seit 1922. Hans-Joachim Denecke (1911–1990), Heidelberg, betreute viele Jahre das Zentralblatt.

In den 1990er-Jahren befand sich die Verlagsbranche insbesondere durch die Digitalisierung in einem Umbruch, den auch einige Traditionsunternehmen wirtschaftlich nicht überstanden. Auch bei Springer stagnierten erstmals seit der Nachkriegszeit die Wachstumsraten. Die Abonnementpreise für Fachzeitschriften mussten stark erhöht werden. Die Produktions‑, Versand- und Marketingkosten stiegen. Um den neuen Herausforderungen zu begegnen, wurde 1996 die Online-Plattform „LINK“ (später „SpringerLink“) aufgelegt, die erstmals die Möglichkeit bot, wissenschaftliche Publikationen online zu lesen und zu erwerben. Bertelsmann erwarb 1998 den Springer-Verlag und gründete die Verlagsgruppe Bertelsmann Springer. Springer war 2002 bereits Weltmarktführer im Bereich der elektronisch verlegten Publikationen.

Dann veräußerte 2003 Bertelsmann die Fachverlagssparte Bertelsmann Springer an die britischen Private-Equity-Gesellschaften Cinven und Candover. Es folgte die Fusion mit dem niederländischen Wissenschaftsverlag Kluwer Academic Publishers (KAP), wodurch der weltweit zweitgrößte Fachverlag „Springer Science + Business Media“ entstand. Für Springer bedeutete der Verkauf eine umfassende Neustrukturierung und Modernisierung. Zur Springer-Verlagsgruppe gehörten im Jahr 2006 insgesamt 70 Einzelverlage mit über 5000 Mitarbeitern in 19 Ländern, die 1450 Zeitschriften und jährlich rund 5000 Buchtitel verlegten. Sämtliche Publikationen des Verlags seit dem Gründungsjahr 1842 wurden 2010 gescannt und online verfügbar gemacht. Schließlich entstand 2015 aus der Fusion von Springer Science + Business Media und dem Großteil von Macmillan Science and Education die neue Gruppe Springer Nature. Das Unternehmen erzielt mit 13.000 Mitarbeitern in über 50 Ländern einen Jahresumsatz von etwa 1,5 Mrd. €. Nach Elsevier ist Springer heute weltweit der zweitgrößte Verlag im Bereich Wissenschaft, Technik und Medizin [86].

Die Zeitschrift *HNO* hat derzeit eine Auflage von etwa 1800 Exemplaren und einen Impact-Faktor von 1,088 (2019), der höchste unter den deutschsprachigen Springer-Medizin-Zeitschriften. Die *European Archives of Oto-Rhino-Laryngology* hatten 2018 einen Impact-Faktor von 1,808 [87].

### Thieme

Nachdem er den Beruf des Buchhändlers erlernte und im Anschluss einige Jahre im Buchhandel in Leipzig, London, Brüssel und Heidelberg arbeitete, erwarb Georg Thieme (1860–1925) mithilfe väterlichen Kapitals den Kasseler Verlag Theodor Fischer sowie dessen Verlagsrechte an medizinischen Inhalten und gründete im Jahr 1886 in Leipzig eine eigene Verlagsbuchhandlung. Als Thieme seinen Fachverlag ins Handelsregister eintragen ließ, war er der erste deutsche Verleger, der sich von Anfang an ausschließlich medizinischen Themen widmen wollte. Wichtigster Umsatzträger war in der Gründerzeit des Verlags der *Reichs-Medicinal-Kalender* mit einem statistischen Jahrbuch, Gesetzessammlungen und neuen Therapeutika, welcher bis nach dem 2. Weltkrieg vertrieben wurde [89].

Thieme übernahm 1887 die 1875 gegründete und bis heute erscheinende *Deutsche medicinische Wochenschrift* (*DMW*), welche sich unter der Redaktion von Dr. Samuel Guttmann innerhalb weniger Jahre insbesondere durch bahnbrechende Beiträge im Bereich der Bakteriologie zu einem wichtigen Umsatzträger des Verlags entwickelte. In diese Zeit fallen Veröffentlichungen von Emil von Behring zur Diphterie oder Shibasaburo Kitasato zum Tetanus. Den größten Einfluss auf die künftige Entwicklung des Thieme-Verlags und die *DMW* hatte jedoch ein vierseitiger Originalbeitrag von Robert Koch, der im November 1890 unter dem Titel „Weitere Mitteilungen über ein Heilmittel gegen Tuberculose“ eingereicht wurde [53]. Die Entwicklung des Tuberkulins löste in der Medizin eine Euphorie über das Deutsche Reich hinaus aus und steigerte Reputation und Auflagenstärke der *DMW* erheblich.

Ein weiterer Meilenstein in der Geschichte des Verlags war die Entdeckung der „X-Strahlen“ des Würzburger Physikers Conrad Wilhelm Röntgen, welche 1896 in der *DMW* nebst der berühmten Röntgenaufnahme der Hand von Röntgens Ehefrau veröffentlicht wurde [41]. Binnen 12 Monaten erschienen in der *DMW* weitere 22 Originalarbeiten zu dieser bahnbrechenden Entdeckung, die den Anstoß zur Entwicklung von Strahlenphysik, Quantentheorie und Relativitätstheorie gab und damit die gesamte klassische Physik revolutionierte, aber auch ganz neue diagnostische und therapeutische Optionen für die Medizin implizierte.

Befeuert wurde die wirtschaftliche Entwicklung aber auch durch die damals sprunghaft gestiegene Zahl von Medizinstudenten und Ärzten. So verdreifachte sich die Zahl der Studenten bis 1888 innerhalb eines Jahrzehnts auf 9000. Um die Jahrhundertwende gehörte die *DMW* neben der *Berliner klinischen Wochenschrift* und *Münchner medizinischen Wochenschrift* bereits zu den wichtigsten deutschen Medizinpublikationen, seinerzeit allerdings nahezu ausschließlich internistisch-bakteriologisch fokussiert. Lediglich ein chirurgisches Opus fand sich unter 78 medizinischen Publikationen des Verlags im Jahr 1900. Als Töchter der Chirurgie waren zu dieser Zeit nur die Augenheilkunde, Frauenheilkunde und HNO-Heilkunde bereits voll entwickelt. Zwar war die *DMW* allen Spezialdisziplinen gegenüber offen, veröffentlicht wurde von diesen jedoch sehr wenig und auch nur dann, wenn es von allgemeinärztlichem Interesse war (vgl. Staehr 1986 [89]).

Der damalige Weltruhm der deutschen Medizin trug zum wirtschaftlichen Erfolg und zur Expansion des Verlags Thieme u. a. durch zahlreiche Übersetzungen und ausländische Zeitschriftenabonnements bei. Das Portfolio wurde sukzessive erweitert, zuerst durch Zukäufe der Verlage Boas/Hesse und Besold, darunter das berühmte Anatomie-Lehrbuch von Rauber und Kopsch. Es gab allerdings auch einen heftigen Verdrängungswettbewerb unter den etablierten und neuen Verlagen im Deutschen Reich, die 1890 bereits 150 medizinische Fachzeitschriften publizierten. Um in diesem Wettbewerb erfolgreich gegen die großen Verlage wie Enke oder Springer zu bestehen, setzte Thieme neben renommierten Autoren auf innovative Themen und neue Techniken. So befassten sich die aufgelegten Lehrbücher intensiv mit den neuen mikroskopischen und endoskopischen Techniken und wurden mit zahlreichen Abbildungen ausgestattet. Das 1893 in Erstauflage erschienene *Lehrbuch der Ohrenheilkunde* von Louis Jacobson enthielt z. B. bereits 330 qualitativ hochwertige Abbildungen. Daneben generierte eine rasch wachsende Zahl von Inseraten für Medizinprodukte in den verlagseigenen Zeitschriften weitere Einnahmen [89].

Zum Ende des 19. Jahrhunderts, nur 14 Jahre nach seiner Gründung, war der Thieme-Verlag ein florierendes mittleres Unternehmen mit 92 lieferbaren Titeln, davon 6 Zeitschriften. Mit Abstand lukrativste Publikation war zu dieser Zeit die *DMW* mit 500.000 verkauften Exemplaren. Vom zweiten wesentlichen Umsatzträger, dem *Reichs-Medicinal-Kalender*, wurden mehr als 10.000 Exemplare jährlich verkauft. Diese Zahlen sind dennoch recht bescheiden, vergleicht man sie z. B. mit der Berliner Buchhandelsfirma Springer, die zu Beginn des 20. Jahrhunderts bereits mehr als 100 Zeitschriftentitel mit einer Auflage von etwa 4 Mio. jährlich vertrieb. Bis zum 1. Weltkrieg expandierte der Verlag weiter und zog zum wiederholten Male in größere Räumlichkeiten. In den Kriegsjahren waren Bücher und Zeitschriften jedoch zwangsbewirtschaftet, sodass von den 11 Zeitschriften im Jahr 1918 nur noch 4 publiziert wurden.

Im Jahr 1919 trat Bruno Hauff (1884–1963) als Teilhaber in den Thieme Verlag ein, welchen er 1925 nach dem Tod Georg Thiemes übernahm [52]. Der Verlag wurde seither als Familienunternehmen geführt. Die Nachkriegsjahre waren von extremer Hyperinflation und Wirtschaftskrisen geprägt, die auch den Thieme-Verlag schwer in Mitleidenschaft zogen. Für einen US-Dollar musste man im November 1923 mehr als 4 Billiarden Deutsche Mark zahlen, weshalb es v. a. durch den Ausbau des internationalen Geschäfts mittels Devisen gelang, allmählich wieder Erlöse zu generieren. Er dehnte das bisher vorwiegend medizinische Verlagsprogramm auf naturwissenschaftliche Gebiete aus, womit es dem Verlag gelang, in der wirtschaftlich schwierigen Zeit der Weimarer Republik weiter zu expandieren.

Die Zeit des Nationalsozialismus und der 2. Weltkrieg gingen auch am Thieme-Verlag aus verschiedenen Gründen nicht spurlos vorbei. Da Bruno Hauffs Ehefrau jüdischer Abstammung war, war die Verlegerfamilie direkt betroffen. Hunderte jüdische Verleger und Autoren wurden durch Säuberungsaktionen ausgeschaltet. Die deutsche Ärzteschaft setzte der nationalsozialistischen Vereinnahmung der Medizin wenig Widerstand entgegen. Ab 1933 erschienen als Folge davon auch in den Publikationen des Thieme-Verlags zunehmend rasse- und militärmedizinische Veröffentlichungen. Die Kriegshandlungen selbst führten spätestens 1944 zum Erliegen der Verlagsaktivitäten, nachdem die Verlagszentrale in Leipzig bei einem Luftangriff zerstört wurde.

Da Leipzig ab Juli 1945 unter sowjetischer Besatzung stehen sollte, erfolgte auf Betreiben der amerikanischen Militärverwaltung zuvor ein Umzug des Verlagssitzes nach Wiesbaden. Zu dieser Zeit war durch die Alliierten das Drucken und Verbreiten von Zeitschriften und Büchern strikt verboten.

Während Verlage wie Springer oder Urban Schwarzenberg bereits Ende 1945 ihre Tätigkeit wieder aufnehmen konnten, erhielt der Thieme-Verlag von der amerikanischen Militärverwaltung erst im April 1946 die erforderliche Lizenz für die Wiederaufnahme der Verlagstätigkeiten. Nach 19 Monaten kriegsbedingter Zwangspause wurde die erste Nachkriegs-*DMW* veröffentlicht, im gleichen Jahr zog der Verlag nach Stuttgart um. Die Verlagsteile in Leipzig wurden von der Sowjetischen Militäradministration sukzessive enteignet und nach Gründung der DDR in den VEB Georg Thieme überführt. Dieser gab sich als Rechtsnachfolger des 1886 gegründeten Verlags aus und publizierte zudem eine Reihe von Vorkriegszeitschriften.

Im Jahr 1948 erschien unter dem Titel *Zeitschrift für Laryngologie, Rhinologie, Otologie und ihre Grenzgebiete* als Organ der Vereinigung Südwestdeutscher Hals-Nasen-Ohrenärzte erstmals eine HNO-Zeitschrift im Thieme-Verlag.

Bruno Hauffs Sohn Günther (1927–2001) wurde 1953 persönlich haftender Gesellschafter des Verlags. In der Folgezeit wuchs das Unternehmen kontinuierlich weiter. Zwischen 1946 und 1963 wurden neben 15 Zeitschriften etwa 1100 Bücher neu oder in neuer Auflage herausgebracht, 480 davon erschienen als Lizenzausgaben fremdsprachlich im Ausland. Allerdings gab es bis zu Beginn der 1960er-Jahre kaum Veröffentlichungen in Spezialgebieten, seinerzeit wurden lediglich 3 HNO-Bücher im Portfolio des Verlags geführt [89].

Eine weitere Akquise gelang 1971 mit der Übernahme des traditionsreichen Enke-Verlags, der heute führend im Bereich tiermedizinischer Fachliteratur ist. Das bis heute benutzte Corporate Design des Thieme-Verlags mit der blau-blau-weißen Trikolore entstand 1972. Seine internationalen Aktivitäten verstärkte Thieme 1979 und gründete eine Tochterfirma in den USA, die Thieme Stratton Inc., heute Thieme Publishers. Die Integration des Hippokrates Verlag erfolgte 1980. Neben der *Rhinologie, Laryngologie, Otologie* erschienen 3 weitere HNO-Zeitschriften im Thieme-Verlag: *Sprache-Stimme-Gehör* seit 1977, *The American Journal of Otology *seit 1979 und *Seminars in Speech, Language and Hearing* seit 1980 (seit 1983 *Hearing* als eigene Zeitschrift). Albrecht Hauff trat 1982 in den Verlag ein und leitet seit 1990 das Unternehmen als persönlich haftender Gesellschafter. In den 1980er- und 1990er-Jahren folgte der Erwerb verschiedener weiterer Unternehmen und Verlagsprogramme, insbesondere des Sonntag-Verlags, des Parey-Verlags, des Karl Demeter-Verlags und des Karl F. Haug-Verlags. Für den Patienten- und Laienmarkt wurde der TRIAS-Verlag gegründet.

Im Zuge der deutschen Wiedervereinigung 1990 wurde der VEB Georg Thieme Verlag, Leipzig, für den symbolischen Preis von 1 DM von der Treuhandanstalt an Thieme, Stuttgart, zurück übertragen. Das ehemalige Leipziger Stammhaus wurde 1992 geschlossen [55]. Im Jahr 1998 erschienen die ersten elektronischen Bücher bei Thieme. Heute stehen das gesamte Lehrbuchprogramm und viele Monografien in der Thieme E‑Book Library als E‑Books zur Verfügung. Mit der eRef ging 2015 eine umfassende medizinische Wissensplattform online [32].

Im Zuge weiterer Verlagszukäufe und inhaltlicher Entwicklungsstrategien wandelte sich der Thieme-Verlag zur Verlagsgruppe. Auf die internationalen Aktivitäten entfallen etwa 25 % des Umsatzes, zur Jahrtausendwende wurde weltweit bereits ein Jahresumsatz von etwa 100 Mio. € generiert. Die diversen Unternehmen und Programme der Verlagsgruppe wurden 2002 innerhalb der neu gegründeten Unternehmenstochter Medizinverlage Stuttgart vereinigt. Die Mitarbeiterzahl stieg weiter kontinuierlich von etwa 600 im Jahr 1990 auf derzeit etwa 1000 an mit einem Jahresumsatz von 162 Mio. € (2018) [12]. Aktuell erscheint die *Laryngo-Rhino-Otologie* monatlich in einer Auflage von 1900 Exemplaren (Impact-Faktor 2019: 0,972), *Sprache-Stimme-Gehör* hat eine monatliche Druckauflage von 3100 Exemplaren (Impact-Faktor 0,3).

### Karger

Siegbert Samuel Karger (1863–1935) gründete 1890 im Alter von 27 Jahren in Berlin den „Verlag von S. Karger“, nachdem er seine Ausbildung zum Buchhändler in Posen (heute Poznan, Polen) durchlief und im Anschluss einige Jahre als Buchhändler tätig war, zuletzt in der Berliner Buchhandlung Stuhr. Kargers vorrangiges Ziel war es, durch eine Sammlung verschiedener Kompendien das Gesamtgebiet der Medizin darzustellen. Das *Geburtshülfliche Vademecum* wurde als erstes Kompendium bereits im Jahr der Verlagsgründung herausgegeben [45]. Erstes Periodikum war die 1893 aufgelegte und bis heute erscheinende *Dermatologische Zeitschrift*. Frühe Reputation erwarb der Verlag u. a. auch durch die Veröffentlichung wissenschaftlicher Beiträge von Oppenheim und Freud. Die erste HNO-Zeitschrift bei Karger erschien 1908 unter dem Titel *Passow-Schaefer Beiträge zur Anatomie, Physiologie, Pathologie und Therapie des Ohres, der Nase und des Halses *[69].

Zu Beginn des 1. Weltkriegs publizierte der Verlag 9 Zeitschriften und veröffentlichte allein 1915 etwa 50 neue Bücher. Die ökonomische Krise der Folgejahre hinterließ auch am Verlag Karger seine Spuren. Kargers Sohn Heinz (1895–1959) trat nach Abschluss seines Wirtschaftsstudiums zu Beginn der 1920er-Jahre in den Verlag ein und übernahm diesen nach dem Tod seines Vaters im Jahr 1935. Bis dahin wuchs das Unternehmen nach wirtschaftlich schwierigen Jahren wieder und listete zu dieser Zeit bereits mehr als 850 Titel [46, 75].

Wie viele andere Verleger jener Zeit waren Samuel Karger und dessen Nachkommen Juden. Auf Druck der Gestapo mussten die zahlreichen jüdischen Wissenschaftler den Kreis der Autoren und Herausgeber verlassen. Um den zunehmenden politischen Repressalien zu entgehen, siedelte der Verlag mit Unterstützung der medizinischen Fakultät Basel im Jahr 1937 in die Schweiz um. Durch die Umstellung des Zeitschriftenkonzepts auf Mehrsprachigkeit konnten anfangs trotz der schwierigen politischen und wirtschaftlichen Situation internationale Märkte erschlossen und neue Abonnenten gewonnen werden. Die Titel der Zeitschriften wurden auf Latein umgestellt, so nannte sich z. B. das *Archiv für Verdauungskrankheiten *fortan *Gastroenterologia* und die *Passow-Schaefer Beiträge* wurden ab 1938 unter dem Titel *Practica oto-rhino-laryngologica* herausgegeben. Es wurden Artikel in deutscher, französischer, englischer und italienischer Sprache veröffentlicht mit Zusammenfassungen in den jeweils anderen Sprachen.

Die Schweiz war während des 2. Weltkriegs durch seine Neutralität zwar von aktiven Kriegshandlungen verschont, jedoch nahezu komplett von der übrigen Welt abgeschnitten. Dem Verlag gelang es nun kaum noch, seine Kunden zuverlässig mit Publikationen zu beliefern und Manuskripte nach Basel zu bekommen, womit ein Großteil der Einnahmen wegbrach. Auf Vermittlung des Schweizer Biochemikers Guggenheim konnten Gelder verschiedener Schweizer Pharmaunternehmen (z. B. Hoffmann-La Roche, Ciba, Sandoz) die Existenz des wirtschaftlich schwer angeschlagenen Verlags sichern [56]. Während des Kriegs produzierte der Verlag auf Halde und konnte diese gelagerten Bestände in der Nachkriegszeit rasch veräußern. Im Jahr 1959 verstarb Heinz Karger, und sein Sohn Thomas (1930–2020) übernahm im Alter von 29 Jahren die Verlagsführung. Im Gegensatz zu vielen anderen Verlagen verzichtete Karger auf interne Fachredaktionen und setzte stattdessen frühzeitig auf ein reines Gutachterverfahren.

Im Rahmen der Internationalisierung wurde ein weltweites Netz von Verlagsvertretungen aufgebaut und Englisch als führende Sprache eingeführt. In Freiburg im Breisgau gründete Karger 1992 einen deutschen Verlagsstandort, der sich primär an niedergelassene Fachärzte im deutschsprachigen Raum richtet. Im Gesamtverlag wurden 1995 noch etwa 10 % der eingereichten Manuskriptseiten auf Deutsch publiziert [48]. Seit 2018 ist Gabriella Karger (1964–) Vorsitzende des Verwaltungsrats des Unternehmens. Die S. Karger AG hat derzeit etwa 240 Mitarbeiter und publiziert neben etwa 100 medizinischen und naturwissenschaftlichen Zeitschriftentiteln jährlich etwa 50 Buchtitel (Jahresumsatz 280 Mio. Dollar) [67]. Die *ORL-Journal for Oto-Rhino-Laryngology, Head and Neck Surgery* hat einen Impact-Faktor von 1,012. Zwischen 1991 und 2003 wurde beim Karger-Verlag noch eine weitere HNO-Zeitschrift, die *Oto-Rhino-Laryngologia nova: europäische Zeitschrift für Praxis, Klinik und Forschung* herausgegeben.

### Urban und Schwarzenberg

Ernst Urban (1838–1923) und Eugen Schwarzenberg (1838–1908) gründeten 1866 in Wien eine Reise- und Versandbuchhandlung. Mit der Übernahme der *Wiener Medizinischen Presse* im Jahre 1876 wurde der Grundstein für den medizinisch-naturwissenschaftlichen Schwerpunkt des Verlags gelegt. Im Jahr 1879 kam der bis heute erscheinende *Deutsche Ärztekalender* heraus. Der 22-jährige Sohn des Firmengründers, Eduard Urban (1875–1953), gründete 1898 in Berlin eine Zweigniederlassung, sein Zwillingsbruder Karl (1875–1930) übernahm später den Wiener Firmensitz. Die Berliner Fachbuchhandlung Oscar Rothacker wurde 1901 übernommen. Im Jahr 1904 erschien die bis heute aufgelegte *Medizinische Klinik*.

Dann übernahm der Verlag 1909 die 1867 gegründete und vom Kommissionsverlag O. Coblentz publizierte *Monatsschrift für Ohrenheilkunde und Laryngo-Rhinologie*. Ab 1910 erschien diese als Organ der österreichischen otologischen, der Wiener laryngologischen und der Münchener laryngo-otologischen Gesellschaft in erweitertem Umfang. Die Zeit des 1. Weltkriegs sowie das anschließende Jahrzehnt waren Jahre der schweren wirtschaftlichen Rezession, der Inflation, des Aufbaus und der erneuten Wirtschaftskrise. Im Jahr 1920 veröffentlichte der Berliner Laryngologe Gustav Killian eine Beschreibung der von ihm weiter entwickelten Schwebelaryngoskopie, welche auf 77 Seiten mit 44 Abbildungen die Laryngologie wesentlich beeinflusste. In dieser Zeit wurde mit dem ab 1925 erschienenen *Handbuch der biologischen Arbeitsmethoden* auch das umfangreichste Werk der Verlagsgeschichte aufgelegt, welches 107 Bände umfasste.

Nach der Machtübernahme der Nationalsozialisten im Jahr 1933 mussten auch bei Urban Schwarzenberg die jüdischen Mitarbeiter aus dem Verlag ausscheiden. Die Werke der jüdischen Autoren konnten bis zum „Anschluss“ Österreichs im Jahr 1938 noch im Wiener Haus erscheinen. Das Berliner Verlagsgebäude wurde 1943 bei einem Bombenangriff zerstört.

Unmittelbar nach dem 2. Weltkrieg erwarb Eduard Urbans Sohn Heinz (1905–1979) das medizinische Buchprogramm des Verlags J. F. Lehmanns, darunter auch den berühmten *Atlas der Anatomie des Menschen* von Sobotta.

Eduard Urban erhielt 1946 von der amerikanischen Militärverwaltung eine Verlagslizenz und gründete eine Filiale in München, welche 1949 zum Hauptsitz des Unternehmens wurde. Der Wiener Verlagszweig konnte erst ab 1954 von den bisherigen Gesellschaftern als GmbH weitergeführt werden. Zum 100. Geburtstag des Verlags, 1966, wurde der 1939 geborene Urenkel des Firmengründers, Michael Urban, Gesellschafter [95]. Eine weitere Niederlassung wurde 1976 in Baltimore/USA gegründet. Rothacker entwickelte sich derweil zu einer der führenden Versandbuchhandlungen für Ärzte. Mit Unterstützung der Fa. Hoffmann-La-Roche erschien 1984 das bis heute aufgelegte „Roche Lexikon Medizin“ [97].

Urban Schwarzenberg wurde 1998 von der Verlagsgruppe Georg von Holtzbrinck übernommen, wenig später erfolgte die Fusion mit dem ebenfalls zu Holtzbrinck gehörenden Verlag Gustav Fischer, das Unternehmen hieß nun Urban Fischer. Im Jahr 1999 übernahm Urban Fischer das Programm des Ullstein Medical Verlags, wodurch Urban Fischer der zweitgrößte medizinische Buchverlag im deutschsprachigen Raum wurde. Holtzbrinck verkaufte 2003 den Urban Fischer Verlag an den niederländischen Wissenschaftsverlag Elsevier. Im Jahr 2018 beschäftigte dieser 7900 Mitarbeiter und veröffentlichte im selben Jahr 470.000 Peer-Review-Artikel, was 18 % des weltweiten Wissenschafts-Outputs entsprach [27]. Elsevier steht dabei unter weltweiter Kritik zahlreicher wissenschaftlicher Institutionen aufgrund seiner Preisgestaltung und der Weigerung, Open-Access-Daten zur Verfügung zu stellen.

### Stahel

Johann Jakob Stahel (1723–1787) gründete bereits 1753 die Stahel’sche königlich-bayerische Hof- und Universitäts-Buch- und Kunsthandlung in Würzburg. Mit Wissen der hochfürstlichen Regierung erwarb er 1763 die Kleyer’sche Universitätsbuchdruckerei in Würzburg. Hierzu musste er als bereits 40-Jähriger eine Buchdruckerlehre durchlaufen, wonach ihm schließlich der Titel eines „Hochfürstlichen Hofbuchhändlers“ verliehen wurde [64]. Der Stahel’schen Buchhandlung wurde 1882 vom Akademischen Senat der Universität Würzburg der Titel einer Universitäts-Buchhandlung verliehen, nachdem sich der Verlag „durch Herausgabe verschiedener Festschriften an der Säcularfeier der Alma Julia Maximilianea in so hervorragender Weise betheiligte“ [64]. Die Hauptrichtung des Verlagsprogramms war ursprünglich katholisch-theologisch geprägt, wandte sich aber in der Mitte des 19. Jahrhunderts den medizinisch-naturwissenschaftlichen und rechtswissenschaftlichen Fachbereichen zu. Bekannte Publikationen waren u. a. *Cannstatt’s Jahresbericht der Medicin* (1851–1865), *Cannstatt’s Jahresbericht der Pharmacie* (1851–1865) und das von Anton von Tröltsch, Adam Politzer und Hermann Schwartze herausgegebene *Archiv für Ohrenheilkunde* (1864–1873) [14, 77]. Von Stahel wurden in der Folgenzeit keine HNO-Periodika mehr publiziert. Die traditionsreiche Buchhandlung wurde am 15. Februar 1992 aus wirtschaftlichen Gründen geschlossen.

### Hirschwald

August Hirschwald (1774–1848) gründete 1816 in Berlin eine Buchhandlung. Er nahm 1826 die verlegerische Tätigkeit auf, und sein Verlag entwickelte sich im Deutschen Reich zu einem der bedeutendsten Medizinverlage. Verlag und Sortiment wurden 1840 getrennt, Letzteres unter der Firma Hirschwald’sche Buchhandlung weitergeführt. Schon sehr früh erfolgte eine Spezialisierung auf die Medizin und Naturwissenschaften. Nach dem Tod August Hirschwalds trat 1848 sein Sohn Ferdinand Hirschwald (1826–1899) als Teilhaber ein, der nun zusammen mit Hirschwalds Neffen Eduard Aber (1810–1899) den Verlag leitete und zur Blütezeit führte. Wichtige Autoren waren u. a. Virchow, Langenbeck, Billroth, Esmarch oder Helmholtz. Bedeutende Zeitschriftengründungen seinerzeit waren die *Berliner medicinische Central-Zeitung*, (1832), die *Berliner klinische Wochenschrift* (1864) sowie die Archive für klinische Chirurgie, Gynäkologie oder Laryngologie (seit 1893) [76]. Eduard Abers Sohn Albert (1842–1920) führte das Unternehmen fort, konnte aber aufgrund wachsender Konkurrenz die wirtschaftliche Stellung des Verlagshauses nicht halten. Während des 1. Weltkriegs kam die Produktion nahezu zum Erliegen, und es fand sich nach Abers Tod im Jahr 1920 kein Nachfolger innerhalb der Familie. Im März 1921 wurden Verlag und Buchhandlung für 175.000 Mark von Springer übernommen (vgl. Sarkowski 1992, S. 245 f. [73]).

### Vogel

Im Jahr 1808 erwarb Friedrich Christian Wilhelm Vogel (1776–1842) nach seiner Ausbildung zum Buchhändler in Leipzig eine Buchhandlung von L. Crusius (1738–1824). Diese ging auf ein schon 1730 von Teubner (1695–1757) in Braunschweig gegründetes Geschäft zurück, welches nach Leipzig verlegt und in ein wissenschaftlich geprägtes Verlagsgeschäft überführt wurde. Crusius übernahm dieses ab 1764 und übergab es schließlich an seinen Mitarbeiter F. C. W. Vogel, der es bis 1836 führte. Vogel gründete neben einer Druckerei ein Kommissions- und Sortimentsgeschäft. Im Jahr 1847 wurde von Wilhelm Vogel (1808–1872), einem Sohn von F. C. W Vogel, der Göttinger Universitätsbuchhandel von Johann Christian Dieterich zugekauft. Carl Lampe-Vischer (1836–1907) übernahm 1862 das Verlagsgeschäft unter Beibehaltung der alten Firma.

Durch sein Interesse für die Medizin wurde das Verlagsprogramm zunehmend mit Werken aus diesem Bereich bestückt. Einer der bekanntesten otologischen Vertreter war der Augen- und Ohrenarzt Anton von Tröltsch (1829–1890). Sein *Lehrbuch der Ohrenheilkunde *erschien 1862 bei Vogel. Die im Jahr 1864 von ihm gemeinsam mit Adam Politzer und Hermann Schwartze gegründete und zunächst bei Stahel verlegte Zeitschrift *Archiv für Ohrenheilkunde* wurde ab 1873 ebenfalls bei Vogel in Leipzig publiziert. der Sohn Carl Friedrich Lampe-Vischer (1864–1937) trat 1890 in das Unternehmen ein und übernahm es 1907 nach dem Tod seines Vaters. Der Gesamtkatalog Vogels wies 1930 knapp 1000 Titel auf, wovon etwa 500 noch lieferbar waren [39]. Seit Beginn des 1. Weltkriegs gab es allerdings nur noch etwa 100 Neuerscheinungen. Lampe-Vischer verkaufte den Verlag 1931 an Springer, da seine Söhne nicht an der Verlagsfortführung interessiert waren. Bis 1940 erschienen insbesondere die Zeitschriften des Verlags F. C. W. Vogel bei Springer noch unter dem alten Verlagsnamen, u. a. das 1864 gegründete *Archiv für Ohren‑, Nasen- und Kehlkopfheilkunde* (vgl. Sarkowski 1992, S. 311 f. [36, 73]).

### Barth

Der in Straßburg geborene Johann Philipp Haug (1747–1784) gründete 1780 in Leipzig einen buchhändlerischen Betrieb ohne spezifisches Verlagsspektrum. Im Jahr 1784 übernahm seine Witwe Catharina Wilhelmina Haug (1755–1799) die Buchhandlung. Johann Ambrosius Barth (1760–1813) ehelichte C. W. Haug, welche ihm den Betrieb überschrieb. Zunehmend fanden wissenschaftliche Werke Aufnahme in das Verlagsprogramm, so beispielsweise ab 1809 die *Annalen der Physik*. Sein Sohn Wilhelm Ambrosius Barth (1790–1851) übernahm, nachdem sein Vater 1813 in den Wirren der Befreiungskriege bei seiner Hilfe in einem Militärlazarett an Spitaltyphus gestorben war, im Alter von 23 Jahren den Verlag zunächst erfolgreich und erweiterte das wissenschaftliche Spektrum. Er beging allerdings Suizid aufgrund finanzieller Schwierigkeiten, worauf dessen Sohn Adolph Ambrosius Barth (1827–1869) 1852 die Buchhandlung übernahm. Adolph Ambrosius Barth starb 1869 ebenfalls an Typhus und hinterließ ein saniertes und florierendes Unternehmen. Sein Bruder Johann Ambrosius Barth (1834–1887) trat anschließend an die Spitze der Firma. Nach dessen Tod wurde der Verlag 1890 an Arthur Meiner (1865–1952), den Sohn eines wohlhabenden Leipziger Kaufmanns, veräußert [88].

Meiner führte das Unternehmen bis zu seinem Tod 1952 und erweiterte das Spektrum u. a. durch den Zukauf von Zeitschriften des Verlags Breitkopf Härtel sowie den Erwerb der Verlage Ambrosius Abel, Leipzig, Quandt Händel, Leipzig, Curt Kabitzsch, Würzburg, Leopold Voss, Hamburg und Hermann Meusser, Berlin. Die bekanntesten Autoren des Verlags waren u. a. Arrhenius, Boltzmann, Einstein, Helmholtz, Hertz, Planck, Röntgen und Schrödinger. Von 1903 bis 1921 erschien bei J. A. Barth das von Brieger und Gradenigo herausgegebene *Internationale Zentralblatt für Ohrenheilkunde* [43].

Meiner war vielfältig aktiv, u. a. 1918–1923 als Vorstand des Börsenvereins der Deutschen Buchhändler in Leipzig, Stadtverordneter (1907–1915), Handelsrichter (1909–1915) sowie im Gewandhausdirektorium und erhielt 1918 von der Universität Gießen die Ehrendoktorwürde. das Verlagshaus in Leipzig wurde 1943 bei einem Bombenangriff stark beschädigt, und die Verlagstätigkeit kam im Laufe des Jahres 1944 zum Erliegen.

Ab Ende 1945 erhielt der Verlag wieder Lizenzen von der sowjetischen Militäradministration zur Fortsetzung seiner Tätigkeit. 1947 wurde in München zur Sicherung der Verlagszukunft ein weiterer J. A. Barth Verlag gegründet, der 1969 an den amerikanischen Verlag Academic Press und 1974 letztlich an Springer ging.

Nach dem Tod Arthur Meiners 1952 übernahm seine Witwe Hertha Meiner (1875–1964) die Geschäfte. Sie verstarb 1964. Die Tochter und Erbin Annemarie Meiner (1895–1985) hatte von München aus allerdings keinen Zugriff auf den Leipziger Barth-Verlag, da die Kommanditistenanteile der in der Bundesrepublik lebenden Brüder Helmut Meiner (1901–1980) und Wolfgang Meiner (1897–1977) von der Staatsbank der DDR treuhänderisch verwaltet wurden. Annemarie Meiner bevollmächtigte 1966 den zuvor eingesetzten Leipziger Geschäftsführer Klaus Wiecke, die Verlagsleitung weiterzuführen. Nach dem Tod aller 3 Meiner-Erben wurde der VEB Johann Ambrosius Barth, Verlag für Medizin, Stomatologie und Naturwissenschaften, Leipzig, von der Staatsbank und der Stadt Leipzig als Erbenvertreter an die Gruppe VE Verlage für Medizin und Biologie/Berlin, Jena, Leipzig, verkauft und in Volkseigentum überführt. Schließlich wurde der Betrieb 1991 von der Treuhandgesellschaft an die Hüthig-Gruppe veräußert, die Barth-Anteile gingen 1999 an die Thieme-Gruppe. Im gleichen Jahr wurde die in Leipzig noch bestehende Außenstelle geschlossen (vgl. Links 2009, S. 102–105 [55], vgl. Wiecke 1980, S. 70 ff. [102]).

### Bergmann

Joseph Friedrich Bergmann (1849–1917) gründete seinen Verlag 1878 in Wiesbaden, wobei er früh die Medizin-Sparte von Christian Wilhelm Kreidel (1817–1890) mit 78 Titeln übernahm, darunter das von Knapp, Mauthner und Moos herausgegebene *Archiv für Augen- und Ohrenheilkunde*, das er bald in das *Archiv für Augenheilkunde *und *Zeitschrift für Ohrenheilkunde* aufteilte. Nach Kreidels Tod 1890 ging der gesamte, 1843 gegründete Verlag mit den Bereichen Naturwissenschaft und Technik an Bergmann, der seinen Neffen Wilhelm Geck am Verlag beteiligte. Nach Bergmanns krankheitsbedingtem Ausscheiden im Jahr 1914 führte Geck den Verlag weiter [7, 72, 78].

Mit dem befreundeten Fritz Springer vereinbarte Bergmann schon frühzeitig, dass der Springer-Verlag nach seinem Tod seine Anteile am Verlag übernehmen solle. Im Jahr 1929 gingen auch die Anteile von Geck an Springer. Bergmann hatte ein umfangreiches Medizinprogramm, das zum Zeitpunkt der Übernahme bedeutender als das des Springer-Verlags war. Bergmanns besondere Stärke lag u. a. in den Fächern Ophthalmologie und Hals‑, Nasen- und Ohrenheilkunde, die bei Springer kaum vertreten waren.

Da Wiesbaden nach dem 1. Weltkrieg bis 1930 von französischen Truppen besetzt wurde, war der Austausch mit den Autoren, Druckereien und Buchhandlungen schwierig, weshalb 1920 der Sitz des Bergmann-Verlags nach München verlegt wurde. Von hier aus entwickelte sich Bergmann verlegerisch weiter und veröffentlichte unter seinem Signet bis 1928 etwa 300 Neuerscheinungen. Seither wurde der Bergmann-Verlag in München unter kleiner Besetzung weitergeführt und betreute im Wesentlichen seine Zeitschriften und die Neuauflagen älterer Titel. Die Auslieferung wurde 1930 nach Berlin verlegt. Im Jahr 1991 hatte Bergmann unter dem Dach von Springer in München 166 Mitarbeiter (vgl. Sarkowski 1992, S. 234 [73]).
